# Niacin, alkaloids and (poly)phenolic compounds in the most widespread Italian capsule-brewed coffees

**DOI:** 10.1038/s41598-018-36291-6

**Published:** 2018-12-14

**Authors:** Donato Angelino, Michele Tassotti, Furio Brighenti, Daniele Del Rio, Pedro Mena

**Affiliations:** 1 Human Nutrition Unit, Department of Food and Drugs, University of Parma, Parma, Italy; 2 School of Advanced Studies on Food and Nutrition, University of Parma, Parma, Italy; 3 Department of Veterinary Science, University of Parma, Parma, Italy

## Abstract

Coffee is one of the most popular beverages worldwide and, nowadays, one of the most practical way for its preparation is by prepacked capsules. The aim of this study was comparing the content in caffeine, trigonelline, *N*-methylpyridinium (NMP), niacin, and chlorogenic acids of 65 different capsule-brewed coffees, commercialised by 5 of the most representative brands in Italy. Coffees were prepared from capsules following manufacturer’s instructions and analysed with an optimized UHPLC-MS/MS method able to assess all these phytochemicals in one single run. Inter-lot and capsule variability were also studied for a subset of coffee capsules. Except for decaffeinated coffees, caffeine amount accounted between 54 and 208 mg/serving. Regular espresso coffees showed higher trigonelline, NMP, and niacin concentrations than large (*lungo*) and decaffeinated samples, with average serving amounts of 17.96, 1.78, and 0.66 mg, respectively. Regarding chlorogenic acids, caffeoylquinic acids were the most relevant ones (20–117 mg/serving). Feruloylquinic acids were quantified between 8 and 50 mg/serving. Coumaroylquinic acids, hydroxycinnamate dimers, caffeoylshikimic acids, and caffeoylquinic lactones were also present at lower concentrations. Multivariate analysis provided comprehensive information on the phytochemical profile of the different types of coffee, showing a great variability among coffees with some brand-related insights. This study supports the need for accurately characterizing espresso coffees while investigating the beneficial effects of coffee on human health.

## Introduction

Coffee is one of the most consumed beverages in the world, prepared from the roasted seeds of *Coffea* plant cultivars^1^. The two main botanical species used for the production of coffee are *Coffea arabica* and *Coffea canephora* var. Robusta, which differ between them for the higher sucrose, lipid, and trigonelline contents of the former with respect to the latter, which in turn shows higher levels caffeine and chlorogenic acids (CGAs)^2^. However, the roasting process, besides modifying the volatile profile, deeply alters the chemical composition of the beans, increasing the production of melanoidins, main contributors of colour and flavour, at the expense of sucrose, aminoacids and CGAs^2^.

Several epidemiological and intervention studies confirmed that the consumption of three to six cups of coffee may have beneficial effects on cardiovascular diseases and diabetes mellitus, decreasing blood pressure, inflammatory markers and blood cholesterol^3–6^. These effects are mainly attributed to the wide spectrum of bioactive compounds contained in coffee beverages, like niacin, the pyridine alkaloids trigonelline and *N*-methylpyridinium (NMP), the purine alkaloid caffeine and to phenolic compounds^7,8^. Caffeine (1,3,7-trimethylxanthine) has been claimed to have no adverse effects in healthy adults when the daily intake is lower than ~400 mg, equivalent to three to six coffee cups^9^. Besides its psychoactive effects, caffeine has been described to be able to increase metabolic rate, perhaps through increased lipid oxidation and thermogenic mechanisms^10^. Trigonelline contributes to the flavour and taste of coffee, and the roasting process leads to the formation of two main trigonelline derivatives, namely NMP and nicotinic acid, a water-soluble B vitamin, also known as niacin. This last compound is highly bioavailable in coffee, more than in other food sources^11^. Concerning their biological effects, trigonelline and its derivatives have been related to anti-diabetic, neuroprotective, and anti-proliferative activities^12^. Coffee also represents one of the major dietary sources of CGAs^13^, which are quinate esters of hydroxycinnamic acids such as caffeic, ferulic, and *p*-coumaric acids, mainly substituted in the 3′, 4′ and 5′ position^14^. Among them, caffeoylquinic acids (CQAs) are the most relevant compounds, followed by feruloylquinic acids, CGA dimers, and other derivatives such as caffeoylquinic lactones and cinnamoylshikimate esters^8^. Generally, CGAs have been endorsed with several biological activities, such as inhibiting reactive oxygen species production, improving endothelial function by modulating nitric oxide production and/or thromboxane activation, and reducing blood LDL-cholesterol levels^15^.

According to the most recent statistics of the International Coffee Organization, about 1.5 billion coffee cups are consumed every day in the world, prepared in several different ways according to the geography and culture of the country (www.ico.org). The most common form in southern Europe, mainly in Italy, is espresso coffee (EC), prepared from roasted and grounded coffee beans. The beverage is prepared by using a coffee machine which, under high pressure, allows the percolation of a limited amount of hot water through a ground coffee compacted powder in a short time, producing a small volume (15–30 mL) of creamy coffee^14^. Among the different coffee machines used for EC preparation, domestic and bar machines have been flanked by portable electric machines which use small sealed plastic capsules or filters, pre-packed with given amounts of coffee powders^16^. Today, a 25% of the total coffee market share in Italy (https://www.statista.com/statistics/693074/market-share-of-coffee-pods-and-capsule-for-offices-in-italy/) has been taken over by several different brands of coffee capsules, with different coffee powder mixtures, characterized by mixes of coffee cultivars, roasting degrees, and production countries, as well as by the occasional presence of aromas or additives. A recent work of our research group investigated the content of caffeine and CGAs of several EC differing for mixture, country, preparation mode and volume of the beverage^14^. A wide difference in caffeine and CGAs, mostly on the basis of different powder mixtures as well as the volume of consumption, was observed, highlighting that “one cup of coffee” might not be a reproducible serving in order to provide information about any bioactive compound^14^.

To date, no studies have investigated comprehensively the main bioactive compounds in coffee capsules. Thus, the present work aimed to characterize the (poly)phenolic and alkaloid profile as well as the niacin content of up to 65 different coffees prepared with coffee capsules belonging to the five most common brands in Italy, in accordance with specific manufacturers’ instructions and specific machines. Capsule variability was also assayed in order to show the variability existing between different lots of the same coffee powder as well as for the coffee machine along the day.

## Materials and Methods

### Materials

Capsules from sixty-five different types of coffee capsules (named 1 to 65) belonging to five different brands (named A to E), together with their relative coffee machine, were purchased on local markets in Parma or through online stores during 2016 (23 capsules, named A1-A23, from brand A; 15 capsules, B24-B38, brand B; 10 capsules, C39-C48, brand C; 10 capsules, D49-D58, brand D; and 7 capsules, E59-E65, brand E). Two lots for each type of capsule were purchased. The type of coffee – regular, large (*lungo*) or decaffeinated EC – and the amount of coffee powder for each capsule is provided in Supplementary Table 1.

3-*O*-Caffeoylquinic acid (3-CQA), 4-*O*-caffeoylquinic acid (4-CQA), 5-*O*-caffeoylquinic acid (5-CQA), caffeine, trigonelline, NMP, and niacin were purchased from Sigma-Aldrich (St. Louis, MO, USA). Acetonitrile and formic acid were also purchased from Sigma-Aldrich. Water for UHPLC analysis was purchased from VWR Chemicals (Fontenay-sous-bois, France).

### Preparation of coffee brews

Two different lots for each type of coffee were considered and, for each lot, two capsules were extracted. Extractions were performed by using the coffee capsule with its relative brand machine, and according to the manufacturer’s instructions in terms of extraction time and coffee volume. Concerning the amount of coffee, brand A and D machines stop automatically for a pre-determined amount of coffee, while machines of the brand B, C and E leave the consumer free to stop the extraction at the desired time. For this reason, the extraction volume for the capsules belonging to these last brands was standardized by calculating the extraction time useful to reach the volume of coffee suggested by the manufacturer. Pressure of extraction was the one set for each brand machine: A, 19 bar; B, 15 bar; C, 15 bar; D, 19 bar; and E, 20 bar (data refers to the manufacturers’ information booklets). Bidistilled water was used for capsule extraction.

Before each coffee extraction, a washing cycle with bidistilled water was carried out to avoid carry-over effects (this was assessed by analysing the caffeine content in the washing water). Two preliminary coffee extractions were performed prior to sample collection, in order to allow the machine to reach a steady extraction temperature. Each brewed coffee was collected in a graduated glass cylinder for volume and temperature measurements.

In order to study the inter-capsule variability, two cycles of ten consecutive extractions (including a washing step between sequential uses) were carried out for a subset of coffee capsules, with at least a type of coffee for each brand, including decaffeinated coffees: 17 (regular), 20 (*lungo*), and 23 (decaffeinated) for brand A; 26 (regular), brand B; 43 (regular), brand C; 54 (regular), brand D; and 62 (regular), brand E. These cycles were performed in two different days.

Aliquots of the brewed coffees were sampled and stored at −80 °C until analysis.

### Liquid chromatography-mass spectrometry (UHPLC-ESI-MS/MS) analysis and method validation

An aliquot of coffee brew was centrifuged at 17,968 *g* for 10 min at 4 °C and generally diluted 1:50 in 0.1% formic acid in water (v:v) prior to UHPLC-ESI-MS/MS analysis. Ten out of the sixty-five samples (A-13, C-43, C-48, C-49, C-50, D-55, D-56, E-59, E-62, E-63) fell outside the calibration curve ranges of caffeine, and, for this reason, they were diluted 100 folds only for caffeine quantification. A method for the analysis of all the considered coffee phytochemicals in a single chromatographic run was developed and validated. Samples were analysed using an Accela UHPLC 1250 equipped with a linear ion trap-mass spectrometer (MS, LTQ XL, Thermo Fisher Scientific Inc., San Jose, CA, USA) fitted with a heated-electrospray ionization (ESI) probe (H-ESI-II; Thermo Fisher Scientific Inc.). Separations were carried out using a XSelect HSS T3 2.5 µm particle size (50 × 2.1 mm, Waters, Milford, MA, USA). Volume injected was 5 µL, column oven was set to 30 °C, and elution flow rate was 0.3 mL/min. The initial gradient was 97% of 0.1% aqueous formic acid and 3% of acetonitrile 0.1% formic acid, reaching 32% acidified acetonitrile at 6.5 min. From 6.5 to 7 min the acidified acetonitrile was increased to 80%, followed by 1.5 min of 80% acetonitrile and then 1.5 min at the start conditions to re-equilibrate the column.

A selective full scan MS^2^ mode analysis was developed to identify and quantify the coffee phytochemicals. The MS worked with capillary temperature at 275 °C, while the source was set at 300 °C. The sheath gas flow was 60 units, while auxiliary gas pressure was set to 5 units. The source voltage was 4 kV. Positive ionization mode was used for the analysis of caffeine, trigonelline, NMP, and niacin, while negative ionization was used for the phenolic compounds. The capillary and tube lens voltages were 3 and 45 V (positive ionization) and −33 and −98 V (negative ionization), respectively. Collision induced dissociation (CID) equal to 30 (arbitrary units) for all the compounds except for trigonelline, NMP, and niacin, where a CID equal to 38 was applied. Pure helium gas was used for CID.

Data processing was performed using Xcalibur software from Thermo Scientific. All compounds were identified by comparison with exact standards, when available, and published mass spectral and chromatographic data. Quantification was carried out in selected reaction monitoring mode by selecting the relative base peak at the corresponding mass to charge ratio (*m/z*), and through external calibration with commercial standards, when available, or with a reference compound selected on the basis of structural similarity. Details on the identification and quantification of the coffee bioactives are presented in Table 1. IUPAC nomenclature has been used for the CGAs.

**Table 1:** Mass spectral characteristics of the main alkaloids, niacin and phenolic compounds in the coffee samples.

N.	Compound	Ion.Mode	Rt^a^ (min)	[M-H]^+^ or [M-H]- (*m/z*)	MS^b^ (*m/z*)	MSI MI level^c^	Quantified as
1	NMP	+	0.48	95	**79**^c^; 83	1	NMP
2	Trigonelline	+	0.45	138	**92**; 94	1	Trigonelline
3	Niacin	+	0.64	124	**106**; 80	1	Niacin
4	Caffeine	+	3.61	195	**138**	1	Caffeine
5	3-CQA	−	3.18	353	**191**;179;135	1	3-CQA
6	4-CQA	−	3.86	353	**173**; 191; **179**	1	4-CQA
7	5-CQA	−	3.76	353	**191**;179	1	5-CQA
8	3-FQA	−	3.72	367	**193**: 191	2	3-CQA
9	4-FQA	−	4.33	367	**173**; 193	2	4-CQA
10	5-FQA	−	4.70	367	**191**;173	2	5-CQA
11	3-CouQA	−	4.20	337	**191**;179	2	3-CQA
12	4-CouQA	−	4.60	337	**173**; 191	2	4-CQA
13	CSA1	−	3.94	335	**179**; 161	2	5-CQA
14	CSA2	−	4.43	335	**173**; 179	2	5-CQA
15	CQL1	−	4.64	335	**161**; 135	2	5-CQA
16	CQL2	−	4.80	335	**161**; 135	2	5-CQA
17	4-FQL	−	5.72	349	**175**; 193	2	4-CQA

*n*-CQA: *n*-*O*-caffeoylquinic acid; CSA: *n*-*O*-caffeoylshikimic acid; *n*-FQA: *n*-*O*-feruloylquinic acid; *n*-FQL: *n*-*O*-feruloylquinic lactone; *n*-CouQA: *n*-*O*-coumaroylquinic acid; CQL: *n*-*O*-caffeoylquinic lactone; NMP: *N*-methylpyridinium. ^a^Rt, retention time; ^c^the fragment ions used for quantification are highlighted in bold; ^c^Metabolite standards initiative (MSI) metabolite identification (MI) levels^31^.

The method was validated for selectivity, calibration curve range, limit of detection (LOD), lower limit of quantification (LLOQ), upper limit of quantification (ULOQ), intra-day and inter-day precision, and accuracy. Method validation was carried out according to Food and Drug Administration (FDA) guidelines^17^. Selectivity was assessed by analysing acidified water (0.1% formic acid) spiked or not with the selected standard compounds at the LLOQ (Supplementary Table 2), and the method was revealed to be highly sensitive. The evaluation of the range of calibration curves was based on data fitting to linear or quadratic regressions, prioritizing linear fitting. Acceptable fitting was estimated by using the coefficient of determination (R^2^). The LOD and LLOQ for each compound were determined as the concentration in which the fragment ion used for quantification showed a signal-to-noise (S/N) ratio ≥3 and ≥10, respectively. The intra-day precision (repeatability) and inter-day precision (semi-reproducibility) of the method, reported as the relative standard deviation (% RSD), was evaluated at the LLOQ of each compound (L1) and at two higher concentration levels (L2 and L3). Each solution was injected randomly three times per day in three different days. The acceptance criteria were RSD <20% for L1 and <15% for both L2 and L3. Accuracy was calculated in terms of recovery rate for the L2 concentration level of each compound, as the ratio between the mean recorded concentration and the spiked concentration, multiplied by 100.

### Statistical analysis

The SPSS statistical package (SPSS Inc., Chicago, IL, USA, version 25) was used. All data were expressed as mean ± SD (n = 4). Principal component analysis (PCA) with varimax was performed to explore the variability among coffee capsules using amount of bioactives per coffee serving as well as the concentration of bioactives in the coffee (mg/mL).

## Results and Discussion

In a recent historical review, it was claimed that “coffee is never ‘just coffee’”^18^, as beans can grow in different parts of the world and be roasted in different ways, coffee can be prepared following several brewing methods and be served in a wide range of sizes. However, in Italy, the espresso coffee consumed in a “bar” is still the most common choice, and the crave to have something similar available at home recently boosted the market of coffee machines that are easy to use, clean and that allow the preparation of coffees that are close to what people can buy in a bar.

To date, to the best of our knowledge, this is the first work fully focused on the characterization of the main alkaloids and phenolic acids in ECs prepared from capsules of the most representative brands in Italy. Most of these brands also commercialize their products worldwide.

It is worth mentioning that the alkaloid and phenolic profiles of ECs prepared by capsules may result different from the ones prepared with moka pots or bar machines. Pressure and temperature of the machines, as well as physico-chemical characteristics of the coffee powders may be different, which makes comparisons with the literature difficult.

### Physical properties of coffees brewed from capsules

For each of the five considered brands, regular size, *lungo* and decaffeinated versions of capsule ECs were present. Concerning the volume of a serving, regular ECs ranged from 19 mL to 53 mL, while *lungo* coffees fell in the range of 90 mL to 200 mL. Decaffeinated coffees had an average volume of 44 mL, with a spike of 90 mL for sample #A-21, which was a *lungo* decaffeinated coffee (Table 2). It should be noted that serving volumes for regular ECs were slightly higher than those found for ECs served at Italians bars (ranging from 13 to 31 mL)^14^.

**Table 2:** Characterization of the espresso coffees from capsules for volume, main alkaloids, niacin and chlorogenic acid contents.

ID Capsule	Volume (mL)	Caffeine (mg/serving)	Trigonelline (mg/serving)	NMP (mg/serving)	Niacin (mg/serving)	Total CQAs (mg/serving)	Other phenolic acids (mg/serving)	Ratio caffeine/total CQAs
A-1	28.0 ± 1.4	72.42 ± 14.55	8.43 ± 0.63	1.44 ± 0.26	0.77 ± 0.10	23.68 ± 0.27	55.47 ± 0.14	2.99 ± 0.07
A-2	29.5 ± 0.7	1.82 ± 0.20	8.38 ± 1.60	1.19 ± 0.05	0.43 ± 0.07	25.99 ± 1.30	44.77 ± 2.24	0.07 ± 0.00
A-3	82.7 ± 5.9	75.09 ± 11.70	29.38 ± 4.72	1.61 ± 0.12	0.55 ± 0.05	63.11 ± 8.81	114.49 ± 0.12	1.20 ± 0.17
A-4	31.5 ± 4.9	63.55 ± 3.94	16.68 ± 2.64	1.18 ± 0.03	0.39 ± 0.01	39.68 ± 1.98	69.27 ± 3.46	1.60 ± 0.08
A-5	30.5 ± 0.7	60.41 ± 0.59	13.17 ± 0.48	1.54 ± 0.25	0.47 ± 0.02	36.26 ± 1.81	58.20 ± 2.91	1.67 ± 0.08
A-6	31.2 ± 2.5	73.92 ± 5.69	10.70 ± 1.27	1.91 ± 0.34	0.51 ± 0.03	29.62 ± 2.44	59.97 ± 0.02	2.50 ± 0.21
A-7	34.0 ± 2.4	58.35 ± 5.95	12.80 ± 1.84	1.03 ± 0.22	0.34 ± 0.01	32.72 ± 2.89	63.59 ± 0.13	1.79 ± 0.16
A-8	31.5 ± 0.7	2.39 ± 0.05	10.18 ± 1.85	1.41 ± 0.09	0.58 ± 0.07	24.05 ± 1.20	50.14 ± 2.51	0.10 ± 0.00
A-9	27.7 ± 0.9	87.24 ± 20.90	7.30 ± 1.35	1.71 ± 0.29	1.01 ± 0.09	19.97 ± 1.40	50.25 ± 0.11	4.38 ± 0.31
A-10	30.2 ± 1.2	59.77 ± 4.81	10.37 ± 0.36	1.79 ± 0.06	0.53 ± 0.03	26.08 ± 4.78	51.35 ± 0.67	2.33 ± 0.43
A-11	92.5 ± 5.2	92.52 ± 2.04	15.32 ± 4.48	2.51 ± 0.13	0.47 ± 0.03	36.40 ± 0.85	103.32 ± 0.20	2.54 ± 0.06
A-12	30.0 ± 1.4	91.74 ± 5.35	13.78 ± 2.32	1.45 ± 0.05	0.57 ± 0.01	40.01 ± 2.00	77.71 ± 3.89	2.29 ± 0.11
A-13	29.0 ± 2.4	135.63 ± 17.08	6.78 ± 1.25	2.08 ± 0.15	1.01 ± 0.11	22.39 ± 3.36	66.09 ± 0.11	6.13 ± 0.92
A-14	94.5 ± 4.2	83.43 ± 0.49	15.71 ± 3.82	2.41 ± 0.01	0.42 ± 0.04	39.34 ± 4.53	97.84 ± 1.22	2.13 ± 0.25
A-15	34.7 ± 0.5	54.91 ± 12.57	12.69 ± 1.74	1.57 ± 0.16	0.56 ± 0.11	33.58 ± 0.79	75.40 ± 0.76	1.68 ± 0.02
A-16	28.0 ± 0.8	96.43 ± 11.64	12.12 ± 0.33	2.16 ± 0.22	0.91 ± 0.01	37.67 ± 1.78	67.12 ± 0.02	2.56 ± 0.12
A-17	28.0 ± 1.4	63.13 ± 8.36	12.55 ± 0.42	1.53 ± 0.28	0.42 ± 0.01	38.78 ± 1.94	57.93 ± 2.90	1.63 ± 0.08
A-18	35.0 ± 2.1	66.05 ± 4.57	16.43 ± 2.24	1.21 ± 0.10	0.35 ± 0.01	46.90 ± 5.47	87.13 ± 0.18	1.42 ± 0.17
A-19	35.0 ± 2.8	63.20 ± 3.76	11.57 ± 0.74	1.58 ± 0.27	0.47 ± 0.08	31.29 ± 1.56	56.13 ± 2.81	2.02 ± 0.10
A-20	95.0 ± 3.2	89.51 ± 1.36	17.53 ± 2.00	2.92 ± 0.31	0.65 ± 0.13	44.45 ± 3.51	102.14 ± 0.57	2.02 ± 0.16
A-21	89.7 ± 0.5	3.63 ± 0.01	20.01 ± 4.84	1.62 ± 0.18	0.57 ± 0.05	54.28 ± 5.02	105.47 ± 0.15	0.07 ± 0.01
A-22	32.7 ± 1.2	58.02 ± 12.73	13.29 ± 1.24	1.47 ± 0.25	0.44 ± 0.01	32.87 ± 1.55	70.74 ± 0.03	1.77 ± 0.08
A-23	31.2 ± 0.9	1.86 ± 0.09	15.68 ± 0.74	0.98 ± 0.07	0.33 ± 0.07	33.74 ± 1.69	51.92 ± 0.42	0.06 ± 0.00
B-24	33.5 ± 1.7	125.35 ± 19.71	17.31 ± 2.94	1.96 ± 0.15	0.78 ± 0.01	51.89 ± 7.23	89.22 ± 0.11	2.49 ± 0.41
B-25	38.7 ± 2.5	142.10 ± 24.62	16.56 ± 3.24	1.24 ± 0.06	0.55 ± 0.08	57.00 ± 2.70	97.11 ± 0.08	2.50 ± 0.12
B-26	48.5 ± 3.8	89.47 ± 7.76	20.55 ± 1.57	1.52 ± 0.24	0.45 ± 0.09	55.34 ± 0.17	84.36 ± 0.05	1.62 ± 0.00
B-27	50.0 ± 3.7	74.81 ± 5.46	12.49 ± 1.94	2.00 ± 0.11	0.55 ± 0.10	30.87 ± 5.40	66.60 ± 0.02	2.46 ± 0.43
B-28	46.7 ± 4.1	2.16 ± 0.21	14.17 ± 3.08	1.77 ± 0.14	0.78 ± 0.09	35.91 ± 6.53	73.01 ± 3.65	0.06 ± 0.01
B-29	51.0 ± 3.5	130.87 ± 9.78	20.60 ± 3.42	2.17 ± 0.15	0.84 ± 0.14	52.94 ± 4.10	93.25 ± 0.16	2.48 ± 0.19
B-30	54.2 ± 7.1	2.92 ± 0.59	13.47 ± 1.70	2.24 ± 0.36	0.90 ± 0.15	36.56 ± 0.62	81.91 ± 0.42	0.08 ± 0.00
B-31	206.3 ± 13.7	130.77 ± 6.99	20.26 ± 1.71	5.88 ± 1.19	1.16 ± 0.17	46.98 ± 2.70	141.33 ± 1.58	2.79 ± 0.16
B-32	183.8 ± 7.5	178.29 ± 11.99	47.32 ± 7.95	2.97 ± 0.58	0.56 ± 0.08	116.68 ± 5.31	223.42 ± 1.89	1.53 ± 0.07
B-33	126.3 ± 13.2	116.07 ± 5.46	20.54 ± 2.12	3.29 ± 0.32	0.73 ± 0.13	47.34 ± 5.72	110.16 ± 1.20	2.47 ± 0.30
B-34	106.3 ± 7.5	185.92 ± 20.67	18.26 ± 2.25	2.92 ± 0.01	0.92 ± 0.05	58.78 ± 3.66	121.40 ± 0.10	3.17 ± 0.20
B-35	31.5 ± 1.2	99.33 ± 19.89	20.83 ± 3.46	1.41 ± 0.03	0.59 ± 0.05	54.66 ± 3.42	81.25 ± 0.03	1.78 ± 0.05
B-36	30.7 ± 2.2	114.63 ± 19.89	12.43 ± 2.96	2.04 ± 0.28	0.93 ± 0.08	33.88 ± 4.04	72.22 ± 0.04	3.41 ± 0.41
B-37	31.2 ± 3.8	107.66 ± 11.02	18.60 ± 2.73	1.46 ± 0.03	0.56 ± 0.07	52.83 ± 6.12	86.82 ± 0.32	2.05 ± 0.24
B-38	47.5 ± 2.3	87.13 ± 2.60	22.33 ± 3.95	1.95 ± 0.22	0.57 ± 0.14	62.88 ± 8.41	98.79 ± 0.03	1.40 ± 0.19
C-39	48.5 ± 0.7	123.17 ± 9.18	26.69 ± 2.54	2.17 ± 0.24	0.73 ± 0.12	86.61 ± 4.33	114.71 ± 5.74	1.42 ± 0.07
C-40	47.7 ± 4.9	1.88 ± 0.16	30.50 ± 0.19	2.06 ± 0.25	0.75 ± 0.16	73.71 ± 0.49	93.59 ± 0.14	0.03 ± 0.00
C-41	46.2 ± 2.6	134.77 ± 12.06	26.49 ± 1.72	2.26 ± 0.13	0.87 ± 0.09	74.26 ± 13.93	109.27 ± 0.14	1.85 ± 0.35
C-42	48.0 ± 2.5	152.59 ± 11.12	14.32 ± 0.36	2.48 ± 0.09	0.93 ± 0.13	50.41 ± 7.66	113.17 ± 0.03	3.06 ± 0.47
C-43	47.5 ± 2.3	200.05 ± 9.64	19.55 ± 3.80	3.02 ± 0.29	0.97 ± 0.06	65.60 ± 1.43	122.89 ± 0.75	3.05 ± 0.07
C-44	100.0 ± 7.1	190.32 ± 5.40	33.47 ± 1.03	3.36 ± 0.20	1.05 ± 0.09	98.97 ± 4.95	156.46 ± 7.82	1.92 ± 0.10
C-45	51.0 ± 1.4	150.20 ± 3.24	23.10 ± 4.04	2.32 ± 0.00	0.62 ± 0.09	68.19 ± 3.41	114.40 ± 5.72	2.20 ± 0.11
C-46	48.0 ± 4.5	157.31 ± 2.10	19.22 ± 1.05	3.04 ± 0.43	1.17 ± 0.15	51.94 ± 1.81	112.05 ± 0.16	3.03 ± 0.11
C-47	49.5 ± 2.6	122.10 ± 9.31	22.00 ± 0.82	2.00 ± 0.35	0.60 ± 0.05	57.63 ± 2.19	101.41 ± 0.02	2.12 ± 0.08
C-48	47.7 ± 3.5	207.99 ± 4.20	16.04 ± 2.90	1.73 ± 0.21	0.75 ± 0.01	69.42 ± 12.62	141.61 ± 0.02	3.05 ± 0.55
D-49	25.0 ± 6.3	117.11 ± 15.60	24.52 ± 1.44	0.81 ± 0.11	0.31 ± 0.03	67.85 ± 10.85	91.17 ± 0.19	1.75 ± 0.28
D-50	22.0 ± 3.4	105.87 ± 2.97	24.48 ± 3.19	1.16 ± 0.01	0.50 ± 0.07	58.02 ± 4.53	81.93 ± 0.03	1.83 ± 0.14
D-51	27.0 ± 2.8	53.87 ± 1.66	28.72 ± 1.11	1.43 ± 0.28	0.66 ± 0.05	53.19 ± 2.66	77.10 ± 3.85	1.01 ± 0.05
D-52	28.5 ± 0.7	4.02 ± 0.36	19.87 ± 0.71	1.65 ± 0.02	0.71 ± 0.00	41.03 ± 2.05	69.32 ± 3.47	0.10 ± 0.00
D-53	132.5 ± 3.5	121.86 ± 10.70	21.10 ± 0.37	3.15 ± 0.27	0.73 ± 0.10	54.88 ± 2.74	108.97 ± 5.45	2.22 ± 0.11
D-54	29.0 ± 0.0	108.95 ± 9.26	15.10 ± 0.10	1.98 ± 0.30	0.59 ± 0.06	50.70 ± 2.54	80.74 ± 4.04	2.15 ± 0.11
D-55	29.5 ± 0.7	134.10 ± 3.89	11.16 ± 0.26	3.43 ± 0.12	1.07 ± 0.01	31.35 ± 1.57	68.40 ± 3.42	4.28 ± 0.21
D-56	27.0 ± 2.8	125.67 ± 0.40	18.72 ± 1.11	1.68 ± 0.25	0.73 ± 0.04	46.41 ± 2.32	78.22 ± 3.91	2.71 ± 0.14
D-57	32.5 ± 2.1	108.73 ± 5.62	33.57 ± 2.25	1.63 ± 0.21	0.47 ± 0.03	75.67 ± 3.78	99.64 ± 4.98	1.44 ± 0.07
D-58	32.0 ± 1.4	103.28 ± 2.16	24.31 ± 1.42	1.19 ± 0.02	0.48 ± 0.02	59.89 ± 2.99	81.66 ± 4.08	1.72 ± 0.09
E-59	33.7 ± 3.9	148.57 ± 27.55	17.21 ± 3.94	1.78 ± 0.06	0.76 ± 0.04	56.40 ± 3.74	103.39 ± 0.07	2.59 ± 0.25
E-60	36.0 ± 3.3	4.57 ± 0.87	32.33 ± 5.99	1.45 ± 0.29	0.68 ± 0.03	62.98 ± 14.90	101.08 ± 0.61	0.07 ± 0.02
E-61	35.0 ± 0.8	107.52 ± 10.62	29.44 ± 7.35	2.19 ± 0.03	0.74 ± 0.05	63.25 ± 11.31	87.19 ± 0.02	1.73 ± 0.31
E-62	35.7 ± 4.0	172.28 ± 40.02	18.99 ± 0.52	2.00 ± 0.10	0.83 ± 0.00	58.00 ± 4.59	109.27 ± 0.05	2.98 ± 0.24
E-63	36.0 ± 5.3	141.98 ± 16.38	19.91 ± 3.96	1.94 ± 0.23	0.82 ± 0.06	61.60 ± 9.53	100.25 ± 0.07	2.33 ± 0.36
E-64	37.5 ± 2.0	127.10 ± 13.97	24.35 ± 4.13	1.89 ± 0.21	0.72 ± 0.01	67.94 ± 9.68	100.93 ± 0.57	1.89 ± 0.27
E-65	35.7 ± 1.2	123.99 ± 16.43	25.06 ± 4.53	1.82 ± 0.38	0.79 ± 0.02	68.50 ± 10.07	105.24 ± 0.75	1.83 ± 0.27

Values are expressed as mean ± SD (n = 4). Legend: CQAs: caffeoylquinic acids; NMP: *N*-methylpyridinium.

The temperatures registered along all the brewed coffees ranged between 60.0 °C to 78.5 °C, without relevant differences among the different ECs and brands (Supplementary Table 3).

### Identification and quantification of niacin, alkaloids and phenolic compounds in capsule coffees

**Figure 1:**
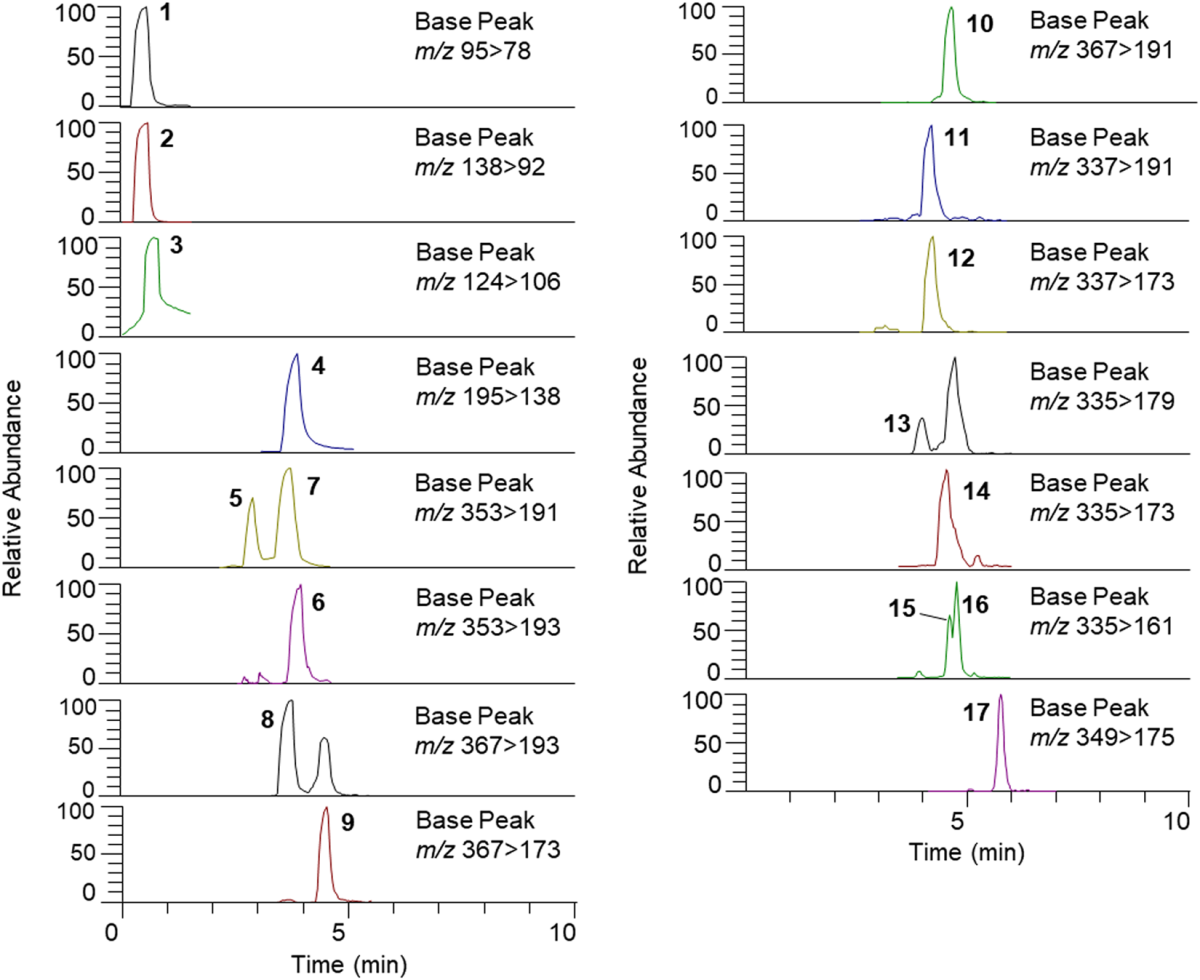
UHPLC-MS/MS profile of capsule-brewed coffees with detection of the considered compounds by SRM. Legend: *m/z* 95 for *N*-methylpyridinium (**1**); *m/z* 138 for trigonelline (**2**); *m/z* 124 for niacin (**3**); *m/z* 195 for caffeine (**4**); *m/z* 353 for caffeoylquinic acids (**5–7**); *m/z* 367 for feruloylquinic acids (**8–10**); *m/z* 337 for coumaroylquinic acids (**11–12**); *m/z* 335 for caffeoylshikimic acids and caffeoylquinic lactones (**13–16**); *m/z* 349 for feruloylquinic lactones (**17**). See Table 1 for further details.

The UHPLC-ESI-MS/MS analysis developed and validated for this study allowed the identification and quantification of up to 17 different compounds in ECs in a single chromatographic run (Fig. 1). Their mass spectral characteristics are showed in Table 1. Interestingly, niacin and very polar pyridine alkaloids trigonelline and NMP, present a good peak resolution under the chromatographic conditions used (Fig. 1). Only the most relevant phenolic compounds, contributing significantly to the total amount of CGAs in coffee, were taken into account in this analysis^19,20^. Besides 3-, 4-, and 5-*O*-caffeoylquinic acids, other phenolics quantified in this study were 3-, 4-, 5-feruloylquinic acids; 3- and 4-*O*-coumaroylquinic acids; caffeoylshikimic acids, caffeoylquinic lactones, and 4-*O*-feruloylquinic lactone. Concerning caffeoylshikimic isomers (CSA1 and CSA2, Table 1), these were tentatively identified as 4-*O*-caffeoylshikimic acid and 3-*O*-caffeoylshikimic acid, by comparing their elution profile and fragmentation patterns with those found by Jaiswal *et al*.^21^. Nevertheless, 3-*O*-caffeoylshikimic acid was not fully confirmed because of the presence of a fragment ion at *m/z* 173, that was not reported by Jaiswal *et al*.^21^. Similarly, caffeoylquinic lactone isomers CQL1 and CQL2 were tentatively identified as 3-*O*-caffeoylquinic lactone and 4-*O*-caffeoylquinic lactone according to Jaiswal *et al*.^21^.

Overall, this validated analytical method allowed a high throughput and accurate fingerprinting of main coffee bioactives, in a unique chromatographic run lasting 10 min. It could be used for further studies assessing objectively the intake of coffee bioactive compounds in epidemiological studies.

Since volumes of ECs changed in relation to the manufacturer’s instruction, results on phytochemical composition have been expressed both as mg per serving and mg/mL (Table 2 and Supplementary Tables 3–5).

### Alkaloid and niacin contents of the capsule espresso coffees

The alkaloids considered in the present work were caffeine, trigonelline and NMP. The caffeine content was highly variable (Table 2). The average amount of caffeine per cup was 108.6 mg/serving for regular ECs and 137.4 mg/serving for *lungo* ECs. Considering individual brands (excluding decaffeinated coffees), ECs belonging to brand C had the highest content of caffeine, (156.0 ± 32.4 mg/serving), while coffees from brand A reached an average content of 73.6 ± 21.3 mg/serving. All the other brands showed an average content of caffeine over 100 mg/serving. Regarding caffeine concentrations in caffeinated coffees, Supplementary Table 3 reports a caffeine range between 0.64 mg/mL and 4.89 mg/mL. The results of the present work are in agreement with those of Albanese *et al*.^16^, who evaluated the caffeine content of coffees brewed from pods by using machines working at pressures comparable to the ones used for this experiment. Authors found amounts of caffeine in pod-brewed coffees (pure Arabica, pure Robusta, and blends) ranging from 2.59 mg/mL to 4.65 mg/mL. Furthermore, our data fell within the range of values found by Andueza *et al*.^22^ under similar conditions of pressure and temperature, reporting an average of caffeine around 2 mg/mL. On the contrary, a recent paper of Jeon *et al*.^23^ showed lower values (<1 mg/mL) in home-made coffees prepared with grounded coffee beans packed in paper filters and extracted with hot water (no information on pressure available). The caffeine content of coffees prepared with bar espresso machines was studied by Caprioli *et al*.^24^. Different temperatures (88 °C, 92 °C and 98 °C), pressures (values between 7 and 11 bar), and types of coffees (pure Arabica, 95% Robusta, and blends) were assessed, and the caffeine content ranged from 111.2 mg to 255.1 mg per serving. These values were slightly higher when compared to the average amount of caffeine in the EC described in the present study (Table 2) which are, however, in agreement with those reported by Ludwig *et al*.^14^, who evaluated several coffee samples brewed with bar espresso machines in Spain, Italy, and Scotland.

Concerning trigonelline, NMP, and niacin, servings of these compounds resulted higher in *lungo* ECs, compared to regular and decaffeinated ECs (Table 2). Trigonelline amounts ranged between 15.32 and 47.32 mg/serving for *lungo* ECs, 8.38 and 33.74 mg/serving for decaffeinated ECs and 6.78 and 17.88 mg/serving for regular ECs. NMP amounts ranged between 1.61 and 6.16 mg/serving for *lungo* ECs, 0.98 and 1.60 mg/serving for decaffeinated ECs and between 0.81 and 3.43 mg/serving for regular ECs. Concerning niacin, *lungo* ECs showed a concentration range of 0.42–1.16 mg/serving, decaffeinated coffee 0.31–0.93 mg/serving and regular ECs 0.31–1.21 mg/serving. However, when concentrations (mg/mL) were considered (Supplementary Table 3), regular ECs resulted in higher trigonelline, NMP, and niacin concentrations, with average values of 0.52 mg/mL, 0.05 mg/mL, and 0.02 mg/mL, respectively. These values almost doubled the respective average values recorded for decaffeinated and *lungo* ECs (Supplementary Table 3). Caprioli *et al*.^24^ also considered the amounts of trigonelline and niacin in coffees brewed with bar espresso machines. Trigonelline values ranged from 22.0 to 49.4 mg/serving for 95% Robusta blends and between 52.5 and 72.7 mg/serving for Arabica, relevantly higher than what reported in Table 2 for most of the ECs. Andueza *et al*. reported trigonelline concentrations close to 1 mg/mL^22^, slightly higher than those found in the present study, although some individual samples (#D-50, #D-51, and #E-61) were over 1 mg/mL (Supplementary Table 3). Regarding niacin amounts, data are in line with those reported by Caprioli *et al*.^24^ and with the USDA Food Composition Databases^25^ for espresso coffee items. Last, in the case of NMP, Lang’s group^26,27^ found average NMP concentrations of 491 nmol/L (~0.046 mg/mL) in brewed coffee, in agreement with our findings (Supplementary Table 3).

### Chlorogenic acids in capsule espresso coffee

The most relevant CGAs in coffee beverages are CQAs, mainly 3-, 4- and 5-CQA. The total amount per serving of these three compounds is shown in Table 2. *Lungo* coffees have the highest average amount of CQAs (60.4 ± 26.6 mg/serving), followed by regular (49.6 ± 16.0 mg/serving) and decaffeinated (43.0 ± 17.4 mg/serving) ECs. Concerning the various brands, brand C coffees revealed to have the highest average content of CQAs (69.5 ± 15.1 mg/serving), followed by E (63.1 ± 4.5 mg/serving), D (53.9 ± 12.7 mg/serving), B (52.5 ± 20.2 mg/serving), and brand A (35.2 ± 10.1 mg/serving). The coffee presenting the highest CQA content was sample #B-32 (116.7 ± 5.3 mg/serving), 3-CQA, 4-CQA and 5-CQA representing 36.1%, 25.7%, and 38.2% of the total CQAs, respectively (Supplementary Table 4). This pattern, registered for most of the tested coffees, was in accordance with literature data for capsule- and filter-brewed coffees^28^, whereas Crozier *et al*.^29^ indicated that 5-CQA was the main CQA in EC, representing about 50% of total CQAs. The mg/serving of total CQAs was also considered by Ludwig *et al*.^14^ for ECs brewed in different countries and from different blends. Data in Table 2 is in line with the CQA amounts per serving reported by these authors for Italian and Scottish ECs, while these were lower with respect to the quantity of CQAs contained in Spanish ECs. Concerning CGA concentrations, our data confirmed the ranges reported by Moeenfard *et al*.^28^, with particular high amounts of 3-CQA and 5-CQA (about 1 mg/mL each) for ECs #D-49 and #D-50 (Supplementary Table 4). These 5-CQA concentrations close to 1 mg/mL, two- to five-fold higher than those recorded for most of the capsule ECs (Supplementary Table 4), were also registered by Andueza *et al*.^22,30^ for a set of blended ECs brewed under different extraction conditions.

Table 2 also shows that ECs belonging to brand C had the highest content in the sum of these phenolics, hitting an average 118.0 ± 18.5 mg/serving, while brand D coffees had the lowest values, with an average of 83.7 ± 12.8 mg/serving. EC #B-32 reached 223.4 ± 1.9 mg/serving, almost double with respect to most of the other coffee samples. Concentration data (mg/mL, Supplementary Tables 4 and 5) were in line with previous results for individual phenolics. These observations back the need for accurately investigating the content in CGAs, and not only in CQAs, of coffee samples. This should be key to comprehensively establish the amount of phenolic compounds provided by a cup of coffee, a point required to further support observational studies with an objective assessment of the intake of these major dietary bioactives^13,14^.

### Degree of roasting

Caffeine/CQA ratio was used as marker of the degree of roasting of the ECs^14^. The caffeine/total CQA ratio, not taken into account for the decaffeinated ECs, was similar among the different brand capsules, ranging from an average of 2.12 for brand D ECs to 2.29 for brand B capsules (Table 2). For instance, considering each type of capsule, EC #D-51 showed the lowest caffeine/total CQA ratio (1.01 ± 0.05), while EC #A-9 showed the highest value (4.38 ± 0.31). Data are in line with those reported by Ludwig *et al*.^14^ for coffees prepared in different European countries and, particularly, the range of caffeine/total CQA ratio was close to the Italian ECs, from 1.3 to 5.3.

### Inter-lot and capsule variability

The inter-lot and capsule variation in the content of main coffee phytochemicals was assessed to better understand how some factors may alter the content in bioactives of capsule ECs. The overall variability for the main compounds between the two lots analysed for each of the 65 coffees fell in the range 10–20% (Table 3).

**Table 3:** Inter-lot variation in the content of main capsule espresso coffee phytochemicals.

ID Capsule	NMP (% CV)	Trigonelline (% CV)	Niacin (% CV)	Caffeine (% CV)	3-CQA (% CV)	4-CQA (% CV)	5-CQA (% CV)
A-1	17.88	7.42	13.21	20.10	2.65	5.45	4.18
A-2	3.78	19.05	15.28	10.96	8.22	15.50	11.04
A-3	7.55	16.06	9.09	15.58	18.37	17.78	7.66
A-4	2.31	15.81	1.66	6.20	9.37	2.97	10.16
A-5	16.33	3.62	4.85	0.97	4.15	8.76	7.04
A-6	17.98	11.92	5.41	7.69	10.22	11.82	2.95
A-7	21.70	14.38	3.40	10.20	19.38	8.60	3.63
A-8	6.71	18.20	11.68	2.17	0.15	0.22	4.82
A-9	16.91	18.55	8.88	23.96	10.69	13.04	3.17
A-10	3.18	3.43	4.73	8.05	21.90	21.34	11.73
A-11	5.15	29.22	5.38	2.20	4.37	2.52	2.04
A-12	3.59	16.84	2.35	5.83	12.80	2.16	0.45
A-13	7.20	18.49	10.79	12.60	21.06	3.56	16.96
A-14	0.54	24.34	8.75	0.58	18.77	16.25	0.03
A-15	10.01	13.72	18.96	22.89	20.79	18.22	18.26
A-16	10.43	2.70	0.99	12.07	4.89	8.60	1.63
A-17	18.50	3.32	2.39	13.24	5.80	11.63	15.93
A-18	8.40	13.64	3.89	6.92	21.10	12.98	0.74
A-19	17.20	6.41	16.64	5.95	2.33	5.99	1.48
A-20	10.72	11.43	20.36	1.52	17.99	11.64	6.16
A-21	10.98	24.19	8.96	0.20	13.26	10.29	4.16
A-22	16.72	9.36	2.98	21.94	15.47	3.68	6.97
A-23	7.52	4.70	22.00	4.94	14.93	4.61	8.01
B-24	7.89	16.97	1.19	15.72	18.94	8.30	11.99
B-25	5.04	19.56	14.29	17.33	16.11	0.36	8.41
B-26	15.66	7.64	20.98	8.68	2.42	12.46	3.81
B-27	5.62	15.53	17.62	7.29	21.87	19.97	8.63
B-28	8.15	21.72	10.99	9.69	21.21	17.08	15.33
B-29	6.91	16.60	16.73	7.47	10.57	5.04	6.07
B-30	16.21	12.59	16.97	20.04	9.58	19.51	5.02
B-31	20.23	8.43	14.27	5.35	13.83	9.89	7.42
B-32	19.39	16.81	14.40	6.72	4.09	13.24	0.84
B-33	9.86	10.31	17.56	4.70	20.85	12.35	3.12
B-34	0.38	12.30	5.27	11.12	4.80	1.94	16.49
B-35	2.15	16.62	9.11	20.03	12.10	16.04	8.41
B-36	13.63	23.83	8.17	17.35	12.65	14.46	8.66
B-37	2.19	14.65	11.99	10.23	5.15	17.88	15.71
B-38	11.44	17.68	24.95	2.98	22.78	13.94	0.77
C-39	11.25	9.53	16.86	7.45	5.83	0.28	3.50
C-40	12.32	0.62	21.83	8.56	3.06	0.83	3.01
C-41	5.62	6.50	9.97	8.94	21.55	22.45	12.38
C-42	3.81	2.49	14.39	7.29	17.61	14.32	12.61
C-43	9.48	19.43	6.06	4.82	2.09	2.81	1.73
C-44	6.03	3.09	8.36	2.84	3.27	14.12	13.46
C-45	0.12	17.47	14.84	2.15	12.60	8.23	7.59
C-46	14.30	5.47	13.11	1.34	1.38	2.27	6.92
C-47	17.33	3.71	7.88	7.63	0.45	9.11	4.84
C-48	12.05	18.07	1.49	2.02	14.09	19.77	21.27
D-49	13.16	5.86	11.41	13.32	21.10	10.66	13.19
D-50	1.03	13.02	13.99	2.81	14.50	6.66	10.29
D-51	19.28	3.85	8.27	3.08	8.17	3.66	7.45
D-52	1.19	3.58	0.61	8.86	14.85	0.68	11.88
D-53	8.73	1.74	14.26	8.78	10.44	2.55	7.83
D-54	15.15	0.66	9.53	8.50	15.71	3.71	1.42
D-55	3.40	2.31	0.80	2.90	4.62	1.58	0.10
D-56	14.88	5.94	6.09	0.32	12.21	16.67	10.32
D-57	13.20	6.69	6.07	5.17	6.93	14.55	6.01
D-58	2.03	5.83	5.13	2.10	0.58	15.76	14.27
E-59	3.30	22.90	5.49	18.55	15.14	1.93	2.10
E-60	20.18	18.52	5.15	19.13	24.72	19.01	25.36
E-61	1.19	24.95	6.93	9.88	21.93	15.85	13.05
E-62	4.95	2.71	0.52	23.23	1.52	8.93	15.96
E-63	11.65	19.89	7.54	11.53	21.78	15.73	4.59
E-64	11.15	16.95	2.08	10.99	15.61	16.52	10.32
E-65	20.80	18.08	3.06	13.25	18.22	20.98	3.50

*n*-CQA: *n*-*O*-caffeoylquinic acid; NMP: *N*-methylpyridinium.

**Figure 2:**
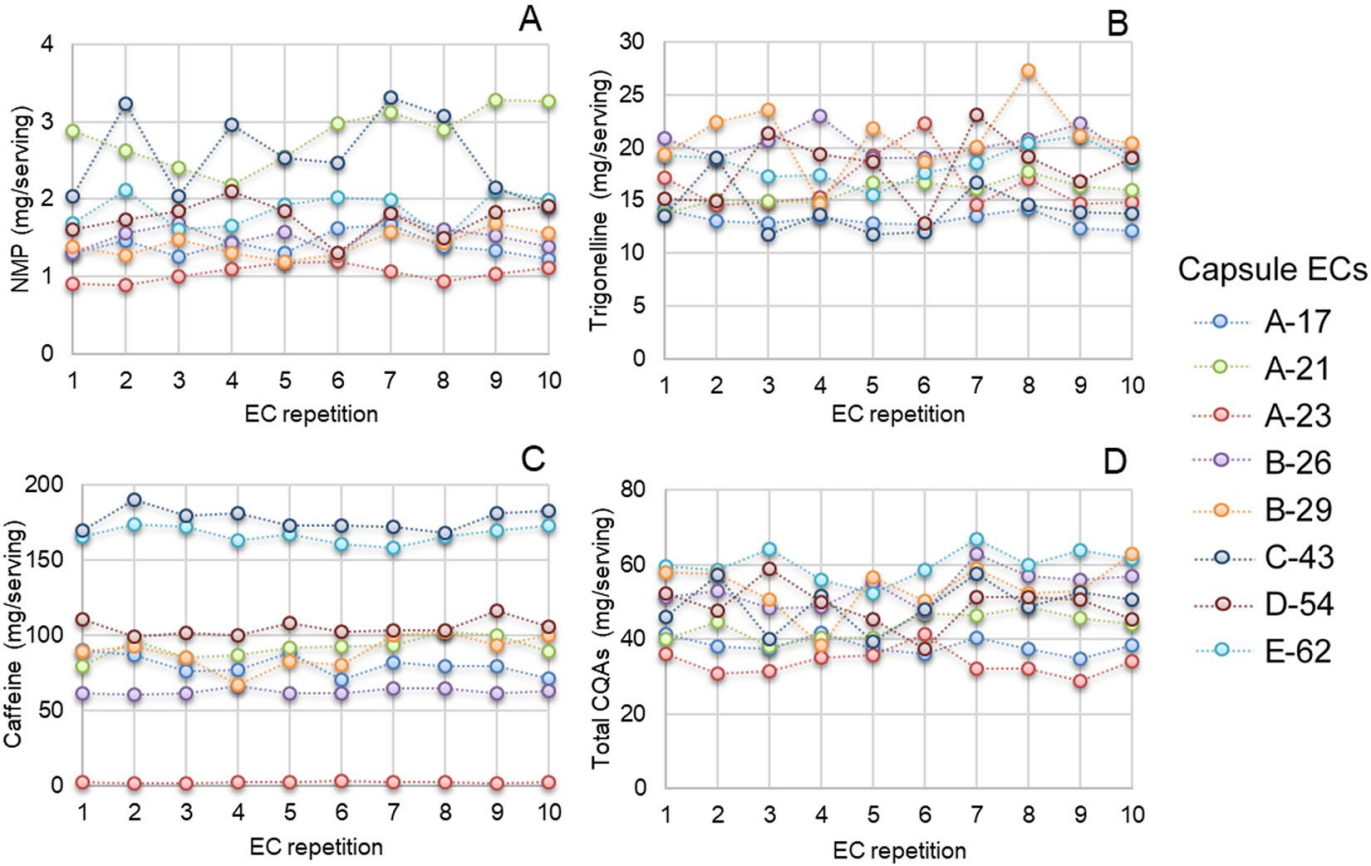
Capsule variability of caffeine (**A**), trigonelline (**B**), *N*-methylpyridinium (**C**) and total caffeoylquinic acids (**D**) of capsule espresso coffee samples. Capsules were representative of regular, *lungo*, and decaffeinated coffees belonging to the five considered brands. CQAs, caffeolylquinic acids; EC, espresso coffee; NMP, *N*-methylpyridinium.

The capsule variability along one day was studied only in a subset of samples for caffeine, trigonelline, NMP, and total CQAs (Fig. 2A–D). Trigonelline and NMP showed the greatest variability, ranging from 8% to 23% and 10% to 21%, respectively. A slightly smaller inter-capsule variability was observed for caffeine, varying between 3% and 20% for the considered coffees. Finally, all the considered brands and capsules showed an average inter-capsule variability along the day lower than 14% for total CQAs.

### Global assessment of the phytochemical profile of the capsule espresso coffees

**Figure 3:**
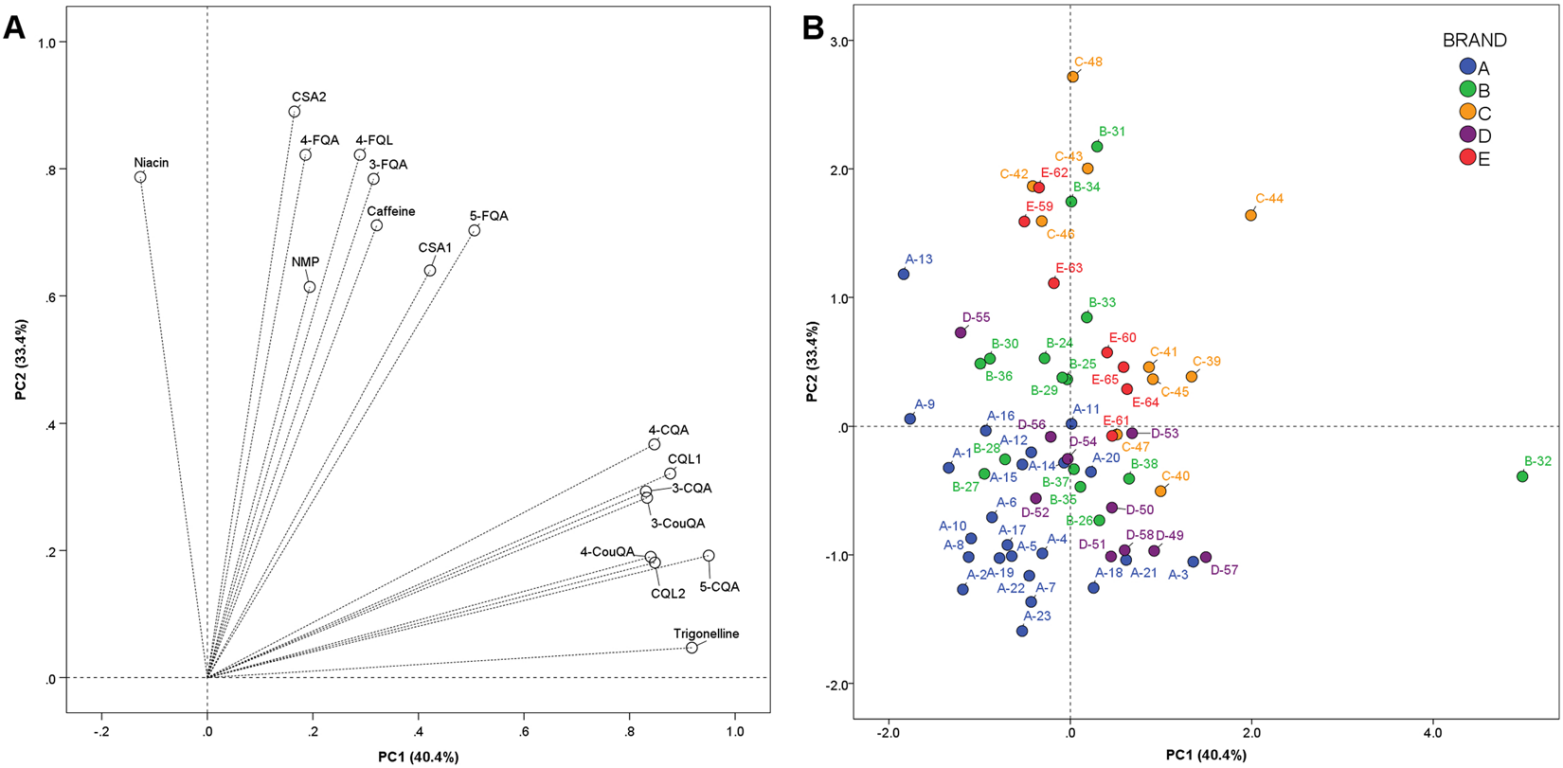
Loading plot (**A**) and score plot (**B**) obtained from the PCA with varimax of the considered bioactive compounds and capsule espresso coffees. Legend: *n*-CQA: *n*-*O*-caffeoylquinic acid; CSA*n*: caffeoylshikimic acid isomer; CQL*n*: caffeoylquinic lactone isomer; *n*-CouQA: *n*-*O*-coumaroylquinic acid; *n*-FQA: *n*-*O*-feruloylquinic acid; *n*-FQL: *n*-*O*-feruloylquinic lactone; NMP: *N*-methylpyridinium.

Multivariate unsupervised PCA, considering the mg/serving of the studied compounds, was carried out in order to better understand the variability in the phytochemical profile of the main coffee capsules commercialised in the Italian market (Fig. 3A). Two principal components (PCs) explained up to 73.8% of the total variability. PC1 accounted for 40.4% of the observed variability and was positively load by trigonelline, the three CQA isomers (3-CQA, 4-CQA, and 5-CQA), the two caffeoylquinic lactones (CQL1 and CQL2), and the two coumaroylquinic acid isomers (3-CouQA and 4-CouQA). PC2 explained a 33.4% of the total variability and had positive loadings from caffeine, niacin, NMP and the phenolic compounds not loading PC1.

Individual scores for each EC revealed interesting insights with regard to the composition of ECs prepared with coffee capsules (Fig. 3B). A pattern where the brand seemed to be a factor conditioning the phytochemical composition of the ECs was observed. Briefly, according to EC scores, most of the brand A coffees had negative values for both PCs, which pointed out that most of the ECs prepared using these capsules are poor in coffee bioactives. Most of the brand B ECs showed neutral scores for PC1, while they displayed a great variability with regard to their scores for PC2. This variability was mainly influenced by the serving size, since ECs #B-31 to B-34 were large – *lungo* – (or very large) ECs. Regarding brand D ECs, these showed positive PC1 scores and negative PC2 ones, which may account for a high content in trigonelline and caffeoylquinic acids and lactones, paralleled to a low content in other alkaloids and phenolic acids. In the case of brands C and E, most of their ECs presented positive scores for both PCs, with a higher variability for PC2. This indicated that they were, comprehensively, the capsule ECs providing the highest amount of coffee bioactives. Contrary to brand B, variability within these ECs was not related to the serving size. This may indicate that the differences in the amount of phytochemicals provided by coffees belonging to brands C and E may be linked to the different types of coffee used for the preparation of the capsules. Finally, while brands C and E offered a great versatility in their product portfolio in terms of coffee bioactives, brands A and B exhibited a homogenous pattern of phytochemicals, despite the putative differences existing in the coffees used for the preparation of each capsule.

An additional PCA, considering the mg/mL of the compounds in coffee samples, was also carried out in order to limit the effect of serving size in the phytochemical profile of the capsules (Supplementary Fig. 1A,B). Similar to results found in Fig. 3A,B, two PCs explained up to 73.8% of the total variability and the main aspects associated with the aforementioned brand-related pattern were observed, with minor variations. Positive PC1 scores and negative PC2 ones have been found for brand D ECs (mainly D-49, D-50, D-51 and D-57), influenced by the high concentrations in trigonelline and caffeoylquinic acids and lactones. On the contrary, brand E ECs were mainly influenced by their concentrations in feruloylquinic acids.

This brand-related pattern was fully unexpected since a high variability in terms of coffee variety, origin, and roasting degree is claimed by each brand when commercialising their different capsules. Consequently, the configuration of a general phytochemical pattern for every brand was not initially hypothesised. Nevertheless, this pattern could be currently attributed to i) the impact that the brewing machine used might have on the extraction of coffee bioactives or ii) the main processing steps used within each company for the preparation of all their capsules. In this regard, Andueza *et al*.^22^ demonstrated by using multivariate analysis the effect of the brewing pressure used in the final profile of coffee components. Further information would be required to confirm the reasons underlying this brand-based pattern in the phytochemical content of ECs produced from capsules.

In conclusion, the alkaloid and phenolic profiles as well as the niacin content of the most representative capsule ECs in the Italian market showed a wide variability both among capsules of the same brand and among different brands. These differences in the composition of ECs prepared by using coffee capsules may be of interest for further studies when a specific set of bioactives is specifically investigated. Moreover, this variability is of note for future studies, since it demonstrates that “coffee is never ‘just coffee’” and that the content of bioactives in a cup of coffee may vary significantly^31^.

## Electronic supplementary material

Supplementary material
