# Supplementary material for: Niacin, alkaloids and (poly)phenolic compounds in the most widespread Italian capsule-brewed coffees
Source: Sci Rep. 2018 Dec 14;8:17874. doi: 10.1038/s41598-018-36291-6 (PMC6294795; doi:10.1038/s41598-018-36291-6)
Supplement: Supplementary file 1 — Supplementary material [file 41598_2018_36291_MOESM1_ESM.pdf]

Niacin, alkaloids and (poly)phenolic compounds in the most widespread Italian capsule-brewed coffees.

Donato Angelino[1], Michele Tassotti[1], Furio Brighenti[1], Daniele Del Rio[1,2,3]*, Pedro Mena[1]

[1]*Human Nutrition Unit, Department of Food and Drugs, University of Parma, Parma, Italy.*

[2]*School for Advanced Studies on Food and Nutrition, University of Parma, Italy.*

[3]*Department of Veterinary Medicine, University of Parma, Italy.*

*** Corresponding author:**
Prof. Daniele Del Rio
University of Parma, Medical School Building A
Via Volturno 39 – 43125 Parma (PR), Italy
Phone: +390521903830; Fax: +390521903832
Email: daniele.delrio@unipr.it

SUPPLEMENTARY INFORMATION

Supplementary Table 1. Powder amount of the coffee capsules used for the coffee preparations.

ID Capsule	Coffee characteristic	Powder amount (g)
**A-1**	Caffeinated	5
**A-2**	Decaffeinated *lungo*	5
**A-3**	Caffeinated *lungo*	5.5
**A-4**	Caffeinated	5
**A-5**	Caffeinated	5
**A-6**	Caffeinated	5
**A-7**	Caffeinated	5
**A-8**	Decaffeinated	5
**A-9**	Caffeinated	5
**A-10**	Caffeinated	5
**A-11**	Caffeinated *lungo*	6
**A-12**	Caffeinated	5
**A-13**	Caffeinated	5
**A-14**	Caffeinated *lungo*	5.5
**A-15**	Caffeinated	5
**A-16**	Caffeinated	5
**A-17**	Caffeinated	5
**A-18**	Caffeinated	5
**A-19**	Caffeinated	5
**A-20**	Caffeinated *lungo*	6
**A-21**	Decaffeinated *lungo*	5.5
**A-22**	Caffeinated	5
**A-23**	Decaffeinated	5
**B-24**	Caffeinated	7.5
**B-25**	Caffeinated	7
**B-26**	Caffeinated	6
**B-27**	Caffeinated	5.2
**B-28**	Decaffeinated	6
**B-29**	Caffeinated	8
**B-30**	Decaffeinated	7
**B-31**	Caffeinated *lungo*	8
**B-32**	Caffeinated *lungo*	10
**B-33**	Caffeinated *lungo*	7
**B-34**	Caffeinated *lungo*	9
**B-35**	Caffeinated	6.5
**B-36**	Caffeinated	7
**B-37**	Caffeinated	7
**B-38**	Caffeinated	7
**C-39**	Caffeinated	7.5
**C-40**	Decaffeinated	7.5
**C-41**	Caffeinated	7.5
**C-42**	Caffeinated	7.5
**C-43**	Caffeinated	7.5
**C-44**	Caffeinated *lungo*	8
**C-45**	Caffeinated	7.5
**C-46**	Caffeinated	7.5
**C-47**	Caffeinated	7
**C-48**	Caffeinated	7.5
**D-49**	Caffeinated	6.7
**D-50**	Caffeinated	6.7
**D-51**	Caffeinated	6.7
**D-52**	Decaffeinated	6.7
**D-53**	Caffeinated *lungo*	6.2
**D-54**	Caffeinated	6.7
**D-55**	Caffeinated	6.7
**D-56**	Caffeinated	6.7
**D-57**	Caffeinated	6.7
**D-58**	Caffeinated	6.7
**E-59**	Caffeinated	7
**E-60**	Decaffeinated	7

**E-61**	Caffeinated	7
**E-62**	Caffeinated	7
**E-63**	Caffeinated	7
**E-64**	Caffeinated	7
**E-65**	Caffeinated	7

Supplementary Table 2. Parameters for quantification of niacin and the main alkaloids and caffeoylquinic acids in coffee capsule samples by UHPLC-MS^2 in SRM mode (MS/MS).

**No.**	**Compound**	**Calibration curve**	**R^2**	**LOD (µg/mL)**	**LLOQ (µg/mL)**	**ULOQ (µg/mL)**	**Precision intra-day (% RSD)**			**Precision inter-day (% RSD)**			**Accuracy (%)**
							**L1**	**L2**	**L3**	**L1**	**L2**	**L3**	
**1**	NMP	$y = -1.4x^2 + 159.6x$	0.987	0.09	0.2	4.7	12.0	9.5	7.7	15.4	13.8	7.9	110.5
**2**	Trigonelline	$y = -56.1x^2 + 21745x$	0.995	<0.01	<0.01	27.4	7.6	4.3	6.5	14.1	13.5	13.4	105.8
**3**	Niacin	$y = 11755x$	0.991	0.01	0.6	12.3	13.3	11.5	6.0	12.9	11.7	9.5	87.4
**4**	Caffeine	$y = 167464x$	0.995	<0.02	0.02	38.8	8.4	6.1	3.2	10.5	6.4	2.3	88.7
**5**	3-CQA	$y = -20.8x^2 + 15381x$	0.998	0.03	0.2	70.9	5.9	7.9	2.1	16.9	12.7	9.7	92.6
**6**	4-CQA	$y = -34.9x^2 + 16234x$	0.999	0.03	0.2	70.9	2.8	10.9	8.9	11.3	10.9	4.5	101.8
**7**	5-CQA	$y = -136.1x^2 + 58230x$	0.991	<0.03	<0.03	70.9	8.5	3.2	2.6	11.1	7.7	6.9	91.5

Legend: LLOQ, Lower Limit of Quantification; LOD, Limit of Detection; n-CQA: *n-O*-caffeoylquinic acid; NMP, *N*-methylpyridinium; RSD, Relative Standard Deviation; ULOQ, Upper Limit of Quantification.

LODs and LLOQs presented as <0.1 indicate a signal-to-noise over 10 (S/N>10), at the indicated concentration. Lower concentrations were not considered for these compounds since all the coffee samples analyze presented values much higher than the LLOQ for these major coffee compounds.

Supplementary Table 3. Temperature and 3-, 4- and 5-caffeoylquinic acid contents in the considered coffee capsules expressed as mg/serving.

ID Capsule	Temperature (°C)	Caffeine (mg/mL)	Trigonelline (mg/mL)	NMP (mg/mL)	Niacin (mg/mL)
**A-1**	70.0±1.4	2.68±0.54	0.30±0.04	0.06±0.00	0.03±0.00
**A-2**	71.5±0.7	0.06±0.01	0.28±0.06	0.04±0.00	0.01±0.00
**A-3**	73.3±1.7	0.91±0.07	0.36±0.03	0.02±0.00	0.01±0.00
**A-4**	71.5±0.7	2.25±0.17	0.46±0.05	0.04±0.01	0.01±0.00
**A-5**	70.5±2.1	1.98±0.07	0.43±0.01	0.05±0.01	0.02±0.00
**A-6**	70.0±1.4	2.11±0.08	0.38±0.07	0.06±0.01	0.02±0.00
**A-7**	70.5±2.1	1.72±0.18	0.36±0.07	0.03±0.01	0.01±0.00
**A-8**	70.5±0.7	0.08±0.00	0.32±0.07	0.04±0.00	0.02±0.00
**A-9**	68.0±2.2	3.17±0.64	0.26±0.05	0.06±0.01	0.04±0.00
**A-10**	70.5±2.4	1.97±0.09	0.34±0.02	0.06±0.00	0.02±0.00
**A-11**	75.3±0.5	1.00±0.01	0.16±0.03	0.03±0.00	0.01±0.00
**A-12**	70.5±2.1	3.06±0.03	0.44±0.07	0.05±0.00	0.02±0.00
**A-13**	68.0±1.8	4.72±0.93	0.25±0.01	0.07±0.01	0.04±0.00
**A-14**	71.8±1.0	0.88±0.00	0.17±0.03	0.03±0.00	0.00±0.00
**A-15**	70.5±1.0	1.53±0.30	0.37±0.05	0.05±0.00	0.02±0.00
**A-16**	69.3±1.5	3.45±0.50	0.43±0.02	0.08±0.01	0.03±0.00
**A-17**	71.0±0.0	2.27±0.41	0.45±0.01	0.05±0.01	0.01±0.00
**A-18**	70.3±2.2	1.89±0.02	0.47±0.09	0.03±0.00	0.01±0.00
**A-19**	70.0±1.4	1.81±0.04	0.33±0.01	0.04±0.00	0.01±0.00
**A-20**	72.8±2.4	0.94±0.01	0.18±0.02	0.03±0.00	0.01±0.00
**A-21**	74.5±0.6	0.04±0.00	0.22±0.04	0.02±0.00	0.01±0.00
**A-22**	71.0±1.2	1.76±0.33	0.41±0.03	0.04±0.01	0.01±0.00
**A-23**	69.8±1.0	0.06±0.00	0.51±0.00	0.03±0.00	0.01±0.00
**B-24**	67.0±2.9	3.74±0.51	0.52±0.10	0.06±0.00	0.02±0.00
**B-25**	70.3±1.7	3.65±0.48	0.38±0.04	0.03±0.00	0.01±0.00
**B-26**	71.8±2.6	1.85±0.01	0.42±0.08	0.03±0.00	0.01±0.00
**B-27**	73.3±2.5	1.50±0.00	0.26±0.02	0.04±0.01	0.01±0.00
**B-28**	71.3±1.7	0.05±0.01	0.31±0.05	0.04±0.01	0.02±0.00
**B-29**	72.5±1.0	2.59±0.30	0.39±0.06	0.04±0.00	0.02±0.00
**B-30**	70.3±1.5	0.05±0.00	0.25±0.06	0.05±0.00	0.02±0.00
**B-31**	75.0±4.7	0.64±0.02	0.10±0.02	0.03±0.00	0.01±0.00
**B-32**	74.4±6.6	0.97±0.09	0.26±0.05	0.02±0.00	0.00±0.00
**B-33**	71.3±7.4	0.93±0.16	0.15±0.02	0.03±0.00	0.01±0.00
**B-34**	78.5±1.3	1.75±0.15	0.17±0.02	0.03±0.00	0.01±0.00
**B-35**	68.8±1.3	3.25±0.74	0.69±0.13	0.04±0.00	0.02±0.00
**B-36**	66.0±3.6	3.75±0.87	0.41±0.07	0.07±0.01	0.03±0.00
**B-37**	68.0±2.5	3.46±0.62	0.58±0.11	0.05±0.00	0.02±0.00
**B-38**	70.8±2.6	1.84±0.11	0.47±0.07	0.04±0.00	0.01±0.00
**C-39**	70.0±0.0	2.54±0.23	0.55±0.06	0.04±0.00	0.02±0.00
**C-40**	65.8±3.0	0.04±0.00	0.65±0.09	0.05±0.00	0.02±0.00
**C-41**	65.3±3.2	2.93±0.46	0.57±0.00	0.06±0.00	0.02±0.00
**C-42**	68.8±1.5	3.19±0.42	0.30±0.01	0.05±0.00	0.02±0.00
**C-43**	67.0±1.2	4.21±0.07	0.41±0.09	0.06±0.01	0.02±0.00
**C-44**	71.0±0.0	1.91±0.08	0.34±0.01	0.03±0.00	0.01±0.00
**C-45**	69.0±0.0	2.95±0.15	0.45±0.07	0.05±0.00	0.01±0.00
**C-46**	67.5±2.5	3.30±0.28	0.40±0.02	0.06±0.00	0.03±0.00
**C-47**	70.5±0.6	2.48±0.33	0.44±0.01	0.04±0.00	0.01±0.00
**C-48**	68.5±3.7	4.37±0.31	0.35±0.07	0.04±0.00	0.02±0.00
**D-49**	59.8±3.5	4.80±0.69	1.02±0.22	0.03±0.01	0.01±0.00
**D-50**	57.5±1.3	4.89±0.81	1.12±0.07	0.05±0.01	0.02±0.00
**D-51**	62.5±0.7	2.00±0.15	1.07±0.07	0.05±0.00	0.02±0.00
**D-52**	61.0±0.0	0.14±0.01	0.70±0.01	0.06±0.00	0.02±0.00
**D-53**	75.0±1.4	0.92±0.11	0.16±0.01	0.02±0.00	0.01±0.00
**D-54**	65.5±0.7	3.76±0.32	0.52±0.00	0.07±0.01	0.02±0.00
**D-55**	64.5±0.7	4.55±0.24	0.38±0.02	0.12±0.01	0.04±0.00
**D-56**	60.5±2.1	4.68±0.51	0.69±0.03	0.07±0.01	0.03±0.00
**D-57**	64.0±4.2	3.35±0.05	1.04±0.14	0.05±0.01	0.01±0.00
**D-58**	64.0±2.8	3.23±0.21	0.76±0.08	0.04±0.00	0.02±0.00
**E-59**	67.3±2.4	4.47±0.47	0.64±0.08	0.05±0.00	0.02±0.00
**E-60**	69.5±1.7	0.13±0.03	0.84±0.11	0.04±0.01	0.02±0.00
**E-61**	64.5±8.3	3.07±0.24	1.00±0.08	0.06±0.00	0.02±0.00
**E-62**	68.3±1.7	4.81±0.58	0.54±0.08	0.06±0.00	0.02±0.00
**E-63**	68.5±2.4	4.02±0.90	0.55±0.05	0.05±0.00	0.02±0.00
**E-64**	67.8±3.2	3.41±0.56	0.66±0.09	0.05±0.01	0.02±0.00
**E-65**	66.5±5.3	3.47±0.49	0.70±0.12	0.05±0.01	0.02±0.00

Values are expressed as mean ± SD (n=4). Legend: NMP, *N*-methylpyridinium.

Supplementary Table 4: 3-, 4- and 5-caffeoylquinic and feruloylquinic acid and 3-, 4-coumaroylquinic acid contents in the considered coffee capsules expressed as mg/serving and mg/mL.

ID Capsule	3-CQA (mg/serving) (mg/mL)	4-CQA (mg/serving) (mg/mL)	5-CQA (mg/serving) (mg/mL)	3-FQA (mg/serving) (mg/mL)	4-FQA (mg/serving) (mg/mL)	5-FQA (mg/serving) (mg/mL)	3-CouQA (mg/serving) (mg/mL)	4-CouQA (mg/serving) (mg/mL)
A-1	**9.69±0.26**	**6.17±0.34**	**7.82±0.33**	**2.50±0.46**	**1.15±0.18**	**4.81±0.71**	**1.90±0.31**	**2.27±0.25**
	0.35±0.01	0.22±0.00	0.28±0.03	0.09±0.02	0.04±0.01	0.17±0.03	0.06±0.01	0.08±0.01
A-2	**12.51±1.03**	**6.15±0.95**	**7.33±0.81**	**2.42±0.24**	**0.99±0.03**	**4.57±0.71**	**1.44±0.21**	**1.68±0.12**
	0.42±0.05	0.21±0.04	0.25±0.03	0.08±0.01	0.03±0.00	0.16±0.03	0.05±0.01	0.06±0.01
A-3	**23.40±4.30**	**14.56±2.59**	**25.15±1.93**	**4.50±0.45**	**1.04±0.03**	**9.28±0.61**	**2.78±0.09**	**6.30±0.56**
	0.28±0.04	0.17±0.01	0.30±0.00	0.05±0.00	0.01±0.00	0.11±0.00	0.03±0.00	0.08±0.01
A-4	**17.41±1.63**	**10.67±0.32**	**11.60±1.18**	**3.25±0.47**	**1.30±0.26**	**7.91±0.17**	**1.95±0.17**	**3.05±0.31**
	0.56±0.14	0.34±0.04	0.41±0.05	0.10±0.00	0.05±0.01	0.25±0.05	0.07±0.01	0.10±0.01
A-5	**15.30±0.64**	**9.16±0.80**	**11.80±0.83**	**2.46±0.26**	**1.26±0.16**	**6.93±0.07**	**2.23±0.37**	**2.30±0.33**
	0.50±0.01	0.30±0.02	0.39±0.02	0.08±0.01	0.04±0.01	0.23±0.00	0.07±0.01	0.08±0.01
A-6	**13.05±1.33**	**7.00±0.83**	**9.57±0.28**	**2.63±0.54**	**1.10±0.07**	**4.61±0.38**	**2.15±0.02**	**2.50±0.28**
	0.44±0.04	0.22±0.01	0.31±0.01	0.08±0.01	0.04±0.00	0.15±0.01	0.07±0.00	0.08±0.01
A-7	**13.58±2.63**	**7.78±0.67**	**11.36±0.41**	**2.36±0.05**	**1.31±0.22**	**5.16±0.34**	**2.11±0.51**	**3.11±0.35**
	0.40±0.08	0.23±0.02	0.34±0.02	0.07±0.00	0.04±0.01	0.15±0.01	0.06±0.01	0.09±0.01
A-8	**10.90±0.02**	**5.44±0.01**	**7.71±0.37**	**2.21±0.05**	**1.30±0.05**	**4.47±0.06**	**1.71±0.16**	**3.17±0.16**
	0.35±0.01	0.17±0.00	0.25±0.02	0.07±0.00	0.04±0.00	0.14±0.01	0.05±0.00	0.10±0.01
A-9	**8.59±0.92**	**5.20±0.68**	**6.18±0.20**	**2.30±0.29**	**1.35±0.01**	**4.10±0.47**	**1.53±0.03**	**1.97±0.03**
	0.31±0.03	0.19±0.02	0.22±0.01	0.08±0.01	0.05±0.00	0.15±0.02	0.05±0.00	0.07±0.00
A-10	**10.65±2.33**	**6.63±1.41**	**8.80±1.03**	**2.04±0.07**	**1.24±0.17**	**5.11±0.21**	**1.81±0.05**	**2.19±0.38**
	0.36±0.07	0.21±0.05	0.29±0.04	0.07±0.00	0.04±0.01	0.17±0.01	0.06±0.00	0.07±0.01
A-11	**12.26±0.54**	**10.75±0.27**	**12.59±0.26**	**3.01±0.26**	**2.14±0.16**	**6.89±0.40**	**3.39±0.36**	**4.58±0.26**
	0.14±0.01	0.11±0.01	0.14±0.01	0.03±0.00	0.02±0.00	0.07±0.00	0.04±0.00	0.05±0.00
A-12	**18.23±2.33**	**10.35±0.22**	**11.43±0.05**	**4.51±0.21**	**1.23±0.03**	**7.77±0.37**	**2.79±0.19**	**3.67±0.10**
	0.61±0.11	0.35±0.01	0.38±0.02	0.15±0.00	0.04±0.00	0.26±0.00	0.09±0.01	0.12±0.00
A-13	**9.57±2.01**	**6.21±0.22**	**6.61±1.12**	**6.23±1.01**	**2.86±0.41**	**9.86±0.57**	**2.07±0.35**	**1.76±0.15**
	0.34±0.03	0.21±0.01	0.23±0.02	0.22±0.03	0.11±0.01	0.34±0.01	0.07±0.01	0.06±0.01
A-14	**16.25±3.05**	**9.14±1.49**	**13.95±0.00**	**3.35±0.38**	**1.82±0.22**	**7.36±0.11**	**2.09±0.04**	**4.26±0.85**
	0.17±0.03	0.10±0.01	0.15±0.00	0.04±0.00	0.02±0.00	0.08±0.00	0.02±0.00	0.04±0.01
A-15	**13.67±2.84**	**9.08±1.65**	**10.83±1.98**	**2.56±0.23**	**1.94±0.12**	**5.88±0.63**	**2.53±0.03**	**3.41±0.80**
	0.39±0.08	0.26±0.05	0.31±0.06	0.07±0.01	0.06±0.00	0.17±0.02	0.07±0.00	0.12±0.00
A-16	**16.18±0.79**	**9.14±0.79**	**12.35±0.20**	**2.87±0.53**	**1.23±0.10**	**7.26±0.41**	**1.73±0.22**	**2.69±0.06**
	0.58±0.01	0.33±0.02	0.44±0.00	0.10±0.01	0.04±0.01	0.26±0.02	0.06±0.01	0.10±0.00
A-17	**16.38±0.95**	**9.95±1.16**	**12.45±1.98**	**3.23±0.29**	**1.18±0.04**	**8.01±0.13**	**1.74±0.07**	**2.09±0.36**
	0.59±0.06	0.36±0.06	0.45±0.09	0.12±0.02	0.04±0.00	0.29±0.02	0.06±0.00	0.08±0.01
A-18	**19.38±4.09**	**11.53±1.50**	**15.99±0.12**	**2.65±0.33**	**0.98±0.07**	**8.04±0.04**	**2.63±0.09**	**3.51±0.49**
	0.45±0.03	0.33±0.06	0.46±0.02	0.07±0.01	0.03±0.00	0.23±0.01	0.08±0.01	0.10±0.01
A-19	**13.84±0.32**	**8.34±0.50**	**9.11±0.14**	**2.33±0.13**	**1.10±0.03**	**6.52±0.41**	**2.11±0.04**	**2.63±0.05**
	0.40±0.02	0.24±0.01	0.26±0.02	0.07±0.00	0.03±0.00	0.19±0.00	0.06±0.00	0.08±0.00
A-20	**17.68±3.18**	**11.09±1.29**	**15.68±0.97**	**2.73±0.40**	**1.13±0.11**	**7.53±0.61**	**2.52±0.10**	**5.34±1.21**
	0.19±0.03	0.12±0.01	0.16±0.01	0.03±0.00	0.01±0.00	0.08±0.01	0.03±0.00	0.05±0.01
A-21	**23.61±3.13**	**10.01±1.03**	**20.65±0.86**	**2.92±0.50**	**1.42±0.15**	**8.03±0.22**	**2.20±0.18**	**5.19±0.15**
	0.26±0.03	0.11±0.01	0.23±0.01	0.03±0.01	0.02±0.00	0.09±0.00	0.02±0.00	0.06±0.00
A-22	**12.93±2.00**	**8.80±0.32**	**11.15±0.78**	**2.40±0.16**	**0.97±0.13**	**6.35±0.75**	**2.09±0.41**	**2.79±0.46**
	0.40±0.07	0.27±0.02	0.34±0.01	0.07±0.00	0.03±0.00	0.19±0.02	0.06±0.01	0.08±0.01
A-23	**14.79±2.21**	**7.94±0.37**	**11.01±0.88**	**2.19±0.17**	**1.00±0.07**	**5.34±0.26**	**1.79±0.04**	**2.77±0.51**
	0.47±0.07	0.25±0.01	0.35±0.02	0.07±0.00	0.03±0.00	0.17±0.01	0.06±0.00	0.09±0.02
B-24	**22.24±4.21**	**14.54±1.21**	**15.10±1.81**	**6.63±0.82**	**1.98±0.37**	**11.75±0.88**	**2.85±0.51**	**3.36±0.67**
	0.67±0.14	0.43±0.05	0.45±0.06	0.19±0.01	0.06±0.01	0.35±0.03	0.09±0.02	0.10±0.02
B-25	**25.66±4.13**	**13.71±0.05**	**17.62±1.48**	**8.24±0.04**	**2.40±0.53**	**16.72±1.26**	**2.42±0.02**	**3.76±0.09**
	0.66±0.13	0.35±0.02	0.45±0.02	0.21±0.01	0.06±0.01	0.43±0.01	0.06±0.00	0.10±0.01
B-26	**24.37±0.59**	**11.92±1.49**	**19.05±0.73**	**4.94±0.83**	**1.73±0.20**	**9.76±1.39**	**2.67±0.28**	**3.90±0.19**
	0.50±0.04	0.25±0.06	0.39±0.02	0.10±0.01	0.03±0.00	0.24±0.00	0.05±0.00	0.08±0.00
B-27	**14.30±3.13**	**7.40±1.48**	**9.17±0.79**	**4.23±0.27**	**1.41±0.03**	**8.84±1.04**	**1.91±0.23**	**2.37±0.35**
	0.28±0.06	0.15±0.03	0.19±0.03	0.09±0.01	0.03±0.00	0.18±0.03	0.04±0.00	0.05±0.00
B-28	**14.69±3.12**	**9.38±1.60**	**11.84±1.81**	**3.97±0.72**	**1.67±0.05**	**7.23±0.11**	**2.49±0.34**	**3.06±0.20**
	0.32±0.05	0.20±0.01	0.25±0.01	0.08±0.01	0.04±0.00	0.16±0.01	0.05±0.00	0.07±0.00
B-29	**22.78±2.41**	**13.34±0.67**	**16.82±1.02**	**4.94±1.06**	**2.15±0.31**	**11.07±1.82**	**2.74±0.60**	**3.63±0.06**
	0.45±0.06	0.26±0.02	0.33±0.03	0.09±0.02	0.04±0.01	0.22±0.04	0.05±0.01	0.07±0.00
B-30	**17.45±1.67**	**7.74±1.51**	**9.52±0.48**	**4.33±0.69**	**2.54±0.29**	**8.96±0.23**	**2.10±0.18**	**4.23±0.62**
	0.36±0.04	0.15±0.03	0.18±0.03	0.08±0.02	0.05±0.00	0.17±0.02	0.04±0.01	0.08±0.00
B-31	**21.01±2.90**	**9.93±0.98**	**16.04±1.19**	**3.91±0.75**	**2.84±0.34**	**9.61±0.98**	**5.09±0.32**	**7.45±0.83**
	0.10±0.02	0.05±0.01	0.08±0.00	0.02±0.00	0.01±0.00	0.05±0.00	0.02±0.00	0.04±0.00
B-32	**42.34±1.73**	**29.86±3.95**	**44.47±0.37**	**8.20±0.03**	**3.47±0.20**	**22.72±1.21**	**6.99±0.25**	**13.35±0.54**
	0.23±0.02	0.16±0.03	0.24±0.00	0.05±0.01	0.02±0.00	0.12±0.01	0.04±0.00	0.07±0.00
B-33	**17.67±3.69**	**11.97±1.48**	**17.70±0.55**	**7.88±1.45**	**2.63±0.03**	**10.74±2.19**	**3.51±0.67**	**6.17±1.24**

	0.14±0.03	0.10±0.02	0.14±0.02	0.07±0.01	0.02±0.00	0.08±0.01	0.02±0.00	0.05±0.00
**B-34**	**27.75±1.33**	**15.14±0.29**	**15.88±2.62**	**11.78±2.04**	**4.07±0.23**	**20.67±1.07**	**3.63±0.48**	**4.63±0.80**
	0.24±0.04	0.13±0.01	0.15±0.02	0.11±0.02	0.04±0.00	0.20±0.01	0.03±0.01	0.04±0.01
**B-35**	**23.95±2.90**	**12.68±2.03**	**18.02±1.52**	**5.22±0.35**	**1.78±0.18**	**11.64±0.12**	**2.94±0.24**	**3.35±0.28**
	0.76±0.06	0.40±0.05	0.57±0.07	0.17±0.00	0.06±0.01	0.37±0.01	0.09±0.00	0.11±0.00
**B-36**	**14.94±1.89**	**8.77±1.27**	**10.16±0.88**	**4.29±0.36**	**2.12±0.04**	**8.17±1.32**	**2.79±0.18**	**3.02±0.29**
	0.48±0.03	0.29±0.02	0.33±0.01	0.14±0.00	0.07±0.00	0.27±0.03	0.09±0.01	0.10±0.00
**B-37**	**23.55±1.21**	**14.27±2.55**	**15.02±2.36**	**5.73±0.97**	**1.93±0.30**	**11.93±0.69**	**2.75±0.52**	**3.09±0.67**
	0.74±0.07	0.43±0.08	0.49±0.06	0.19±0.03	0.06±0.00	0.39±0.01	0.09±0.01	0.10±0.02
**B-38**	**27.05±6.16**	**14.96±2.09**	**20.86±0.16**	**4.35±0.86**	**1.24±0.16**	**11.06±0.45**	**3.38±0.19**	**4.32±0.25**
	0.57±0.12	0.31±0.03	0.44±0.01	0.09±0.02	0.03±0.00	0.23±0.00	0.07±0.00	0.09±0.00
**C-39**	**37.61±2.19**	**21.14±0.06**	**27.86±0.98**	**8.30±1.49**	**2.01±0.12**	**14.08±1.68**	**5.28±0.92**	**3.96±0.01**
	0.78±0.03	0.44±0.01	0.57±0.01	0.17±0.03	0.04±0.00	0.29±0.03	0.11±0.02	0.08±0.00
**C-40**	**33.98±1.04**	**16.72±0.14**	**23.00±0.69**	**5.99±0.16**	**1.88±0.20**	**13.50±0.28**	**4.62±0.22**	**4.20±0.12**
	0.72±0.07	0.35±0.04	0.49±0.08	0.13±0.02	0.04±0.01	0.28±0.03	0.10±0.02	0.09±0.01
**C-41**	**31.32±6.75**	**18.47±4.15**	**24.48±3.03**	**6.06±1.01**	**1.95±0.16**	**13.67±3.04**	**3.92±0.66**	**4.54±0.28**
	0.67±0.10	0.40±0.06	0.53±0.03	0.13±0.01	0.04±0.01	0.29±0.05	0.08±0.01	0.10±0.01
**C-42**	**21.55±3.80**	**13.28±1.90**	**15.57±1.96**	**7.85±0.18**	**3.44±0.17**	**16.05±0.81**	**3.14±0.34**	**4.42±0.36**
	0.45±0.05	0.28±0.02	0.32±0.02	0.16±0.01	0.07±0.01	0.33±0.00	0.07±0.00	0.09±0.00
**C-43**	**27.66±0.58**	**18.49±0.52**	**19.45±0.34**	**9.92±0.42**	**3.00±0.07**	**21.13±3.19**	**3.71±0.02**	**4.81±0.38**
	0.58±0.00	0.39±0.00	0.41±0.00	0.21±0.00	0.06±0.00	0.45±0.05	0.08±0.00	0.10±0.01
**C-44**	**40.60±1.33**	**23.61±3.33**	**34.75±4.68**	**11.05±1.17**	**2.75±0.00**	**18.83±1.96**	**5.54±0.19**	**5.49±0.52**
	0.41±0.04	0.24±0.02	0.35±0.02	0.11±0.00	0.03±0.00	0.19±0.03	0.06±0.01	0.06±0.01
**C-45**	**27.31±3.44**	**18.29±1.51**	**22.60±1.72**	**6.11±0.70**	**1.39±0.03**	**14.49±1.27**	**4.43±0.71**	**4.52±0.55**
	0.53±0.05	0.36±0.02	0.44±0.02	0.12±0.02	0.03±0.00	0.28±0.02	0.09±0.01	0.09±0.01
**C-46**	**21.69±0.30**	**12.61±0.29**	**17.64±1.22**	**7.48±0.15**	**2.26±0.15**	**14.82±1.61**	**3.28±0.39**	**3.52±0.67**
	0.47±0.03	0.27±0.01	0.38±0.00	0.16±0.01	0.05±0.00	0.31±0.07	0.07±0.00	0.08±0.01
**C-47**	**23.00±0.10**	**14.48±1.32**	**20.15±0.97**	**5.98±1.00**	**1.89±0.04**	**14.33±1.06**	**3.61±0.58**	**4.30±0.13**
	0.47±0.03	0.29±0.01	0.41±0.00	0.12±0.01	0.04±0.00	0.29±0.00	0.06±0.01	0.09±0.01
**C-48**	**25.82±3.64**	**19.60±3.87**	**24.00±5.11**	**15.86±1.45**	**4.59±0.24**	**29.39±5.68**	**2.70±0.45**	**4.40±0.56**
	0.56±0.08	0.41±0.06	0.50±0.08	0.33±0.01	0.10±0.00	0.61±0.09	0.06±0.01	0.09±0.01
**D-49**	**28.95±6.11**	**15.44±1.65**	**23.45±3.09**	**4.87±1.14**	**1.18±0.01**	**12.22±2.65**	**3.11±0.09**	**3.87±0.90**
	1.18±0.08	0.54±0.05	0.96±0.14	0.20±0.01	0.05±0.01	0.49±0.03	0.14±0.02	0.16±0.01
**D-50**	**24.69±3.58**	**14.64±0.98**	**18.69±1.92**	**4.05±0.08**	**1.52±0.07**	**9.36±0.46**	**3.42±0.17**	**3.52±0.40**
	1.13±0.05	0.66±0.15	0.86±0.08	0.19±0.03	0.06±0.01	0.43±0.06	0.16±0.02	0.16±0.01
**D-51**	**23.10±1.89**	**13.34±0.49**	**16.74±1.25**	**2.22±0.22**	**0.91±0.03**	**6.66±0.04**	**3.29±0.56**	**3.45±0.19**
	0.86±0.16	0.50±0.03	0.63±0.11	0.08±0.00	0.03±0.00	0.25±0.03	0.14±0.01	0.13±0.01
**D-52**	**17.03±2.53**	**10.61±0.07**	**13.39±1.59**	**3.88±0.47**	**1.31±0.25**	**8.46±0.46**	**2.90±0.32**	**2.88±0.07**
	0.60±0.07	0.37±0.01	0.47±0.04	0.14±0.02	0.05±0.00	0.30±0.01	0.10±0.01	0.10±0.00
**D-53**	**24.42±2.55**	**11.65±0.30**	**18.82±1.47**	**3.86±0.04**	**1.19±0.08**	**10.68±1.31**	**3.97±0.46**	**5.98±0.24**
	0.18±0.01	0.09±0.00	0.14±0.01	0.03±0.00	0.01±0.00	0.08±0.01	0.03±0.00	0.05±0.00
**D-54**	**20.42±3.21**	**13.72±0.51**	**16.56±0.23**	**3.06±0.30**	**1.66±0.08**	**8.66±1.45**	**3.21±0.34**	**3.63±0.05**
	0.70±0.11	0.47±0.02	0.57±0.01	0.11±0.01	0.06±0.00	0.30±0.05	0.11±0.01	0.13±0.00
**D-55**	**13.14±0.61**	**8.13±0.13**	**10.08±0.01**	**3.21±0.32**	**1.73±0.04**	**5.98±0.27**	**2.53±0.42**	**2.91±0.37**
	0.45±0.03	0.28±0.00	0.34±0.01	0.11±0.01	0.06±0.00	0.20±0.00	0.09±0.02	0.10±0.01
**D-56**	**19.51±2.38**	**12.72±2.12**	**14.19±1.46**	**4.39±0.47**	**1.51±0.24**	**8.81±0.22**	**3.51±0.55**	**2.76±0.48**
	0.72±0.01	0.47±0.03	0.53±0.00	0.16±0.00	0.06±0.00	0.33±0.03	0.13±0.01	0.10±0.01
**D-57**	**33.21±2.30**	**16.44±2.39**	**26.01±1.56**	**3.53±0.15**	**1.42±0.16**	**10.57±0.30**	**3.68±0.51**	**5.05±0.03**
	1.02±0.00	0.50±0.04	0.80±0.00	0.11±0.00	0.04±0.01	0.33±0.03	0.11±0.01	0.16±0.01
**D-58**	**26.14±0.15**	**13.76±2.17**	**19.99±2.85**	**4.63±0.51**	**1.64±0.06**	**9.63±0.34**	**3.44±0.49**	**3.68±0.05**
	0.82±0.04	0.43±0.09	0.63±0.12	0.15±0.02	0.05±0.00	0.30±0.02	0.11±0.02	0.12±0.00
**E-59**	**25.05±3.79**	**15.08±0.29**	**16.27±0.34**	**11.79±0.70**	**3.74±0.42**	**19.64±2.69**	**1.92±0.21**	**2.98±0.36**
	0.80±0.13	0.45±0.05	0.49±0.04	0.35±0.02	0.11±0.02	0.58±0.02	0.06±0.01	0.09±0.00
**E-60**	**31.95±7.90**	**13.67±2.60**	**17.36±4.40**	**9.36±2.17**	**4.13±0.18**	**17.45±4.15**	**3.16±0.69**	**3.52±0.69**
	0.82±0.10	0.39±0.07	0.46±0.08	0.26±0.05	0.11±0.00	0.45±0.07	0.08±0.01	0.10±0.02
**E-61**	**30.06±6.59**	**13.96±2.21**	**19.23±2.51**	**3.94±0.43**	**1.52±0.06**	**10.37±2.01**	**3.72±0.64**	**3.43±0.31**
	0.72±0.01	0.40±0.07	0.55±0.08	0.11±0.02	0.04±0.00	0.30±0.06	0.11±0.01	0.10±0.01
**E-62**	**24.61±0.37**	**15.86±1.42**	**17.53±2.80**	**10.76±0.57**	**3.78±0.65**	**17.99±1.86**	**2.28±0.26**	**3.32±0.27**
	0.70±0.07	0.47±0.04	0.51±0.01	0.30±0.02	0.11±0.00	0.51±0.01	0.06±0.00	0.10±0.01
**E-63**	**28.51±6.21**	**16.22±2.55**	**16.87±0.77**	**8.87±0.66**	**2.34±0.17**	**17.61±0.61**	**1.94±0.12**	**3.17±0.30**
	0.79±0.09	0.45±0.02	0.47±0.03	0.25±0.01	0.07±0.00	0.50±0.03	0.06±0.00	0.09±0.00
**E-64**	**29.40±4.59**	**18.01±2.97**	**20.53±2.12**	**5.92±1.40**	**1.66±0.17**	**14.96±1.76**	**3.94±0.07**	**4.06±0.56**
	0.79±0.09	0.47±0.06	0.55±0.03	0.16±0.02	0.04±0.00	0.40±0.02	0.11±0.01	0.11±0.01
**E-65**	**32.96±6.01**	**16.13±3.38**	**19.41±0.68**	**6.52±0.93**	**1.75±0.40**	**15.08±1.32**	**3.89±0.52**	**3.73±0.16**
	0.92±0.16	0.45±0.09	0.54±0.01	0.18±0.02	0.05±0.01	0.42±0.03	0.11±0.02	0.10±0.00

Values are expressed as mean ± SD (n=4). Legend: *n*-CQA: *n*-*O*-caffeoylquinic acid; *n*-CouQA: *n*-*O*-coumaroylquinic acid; *n*-FQA: *n*-*O*-feruloylquinic acid.

Supplementary Table 5. Main caffeoylshikimic and caffeoylquinic acids and feruloyl quinic lactone contents in the considered coffee capsules expressed as mg/serving and mg/mL.

ID Capsule	CSA1 (mg/serving) (mg/mL)	CSA2 (mg/serving) (mg/mL)	CQL1 (mg/serving) (mg/mL)	CQL2 (mg/serving) (mg/mL)	4-FQL (mg/serving) (mg/mL)
**A-1**	**3.31±0.77**	**3.92±0.37**	**18.72±0.87**	**14.44±2.26**	**2.33±0.23**
	0.14±0.01	0.14±0.01	0.67±0.07	0.55±0.06	0.08±0.01
**A-2**	**2.51±0.22**	**2.52±0.41**	**15.91±0.70**	**11.22±0.86**	**1.52±0.27**
	0.09±0.01	0.09±0.02	0.54±0.04	0.38±0.04	0.06±0.00
**A-3**	**4.46±0.40**	**3.51±0.44**	**41.88±6.74**	**37.69±5.54**	**2.96±0.29**
	0.05±0.01	0.04±0.00	0.50±0.05	0.39±0.05	0.04±0.01
**A-4**	**3.42±0.09**	**3.17±0.07**	**25.01±0.72**	**17.88±0.71**	**2.33±0.03**
	0.11±0.01	0.10±0.02	0.81±0.15	0.57±0.07	0.08±0.01
**A-5**	**2.40±0.27**	**3.09±0.16**	**20.73±1.70**	**15.00±0.85**	**1.80±0.07**
	0.08±0.01	0.10±0.00	0.68±0.04	0.49±0.02	0.06±0.00
**A-6**	**3.88±0.29**	**3.30±0.52**	**19.60±0.28**	**18.37±4.92**	**1.82±0.18**
	0.12±0.02	0.11±0.02	0.63±0.04	0.51±0.07	0.06±0.01
**A-7**	**2.92±0.10**	**2.27±0.33**	**23.23±0.73**	**19.22±3.85**	**1.99±0.24**
	0.09±0.00	0.07±0.01	0.69±0.03	0.57±0.11	0.06±0.01
**A-8**	**2.40±0.43**	**3.20±0.05**	**17.23±2.98**	**12.71±1.99**	**1.74±0.23**
	0.08±0.01	0.10±0.00	0.55±0.08	0.40±0.05	0.06±0.01
**A-9**	**3.04±0.66**	**4.75±0.39**	**17.08±0.26**	**12.24±1.50**	**1.97±0.37**
	0.09±0.01	0.17±0.01	0.62±0.02	0.44±0.06	0.07±0.01
**A-10**	**3.04±0.53**	**2.91±0.32**	**16.63±2.17**	**15.25±2.64**	**1.59±0.30**
	0.10±0.02	0.10±0.01	0.55±0.05	0.51±0.08	0.05±0.01
**A-11**	**5.75±0.14**	**5.21±0.58**	**37.34±2.35**	**31.24±2.59**	**3.92±0.84**
	0.06±0.00	0.06±0.00	0.41±0.01	0.34±0.04	0.04±0.01
**A-12**	**4.04±0.34**	**5.46±0.07**	**25.40±0.13**	**19.80±0.97**	**3.04±0.04**
	0.13±0.00	0.18±0.01	0.85±0.04	0.66±0.00	0.10±0.01
**A-13**	**3.49±0.36**	**4.95±0.43**	**17.95±2.65**	**12.51±1.46**	**4.49±0.51**
	0.12±0.00	0.17±0.00	0.62±0.04	0.43±0.02	0.16±0.03
**A-14**	**4.32±0.98**	**4.35±0.88**	**34.74±4.20**	**30.50±6.19**	**4.19±0.78**
	0.05±0.00	0.04±0.01	0.37±0.05	0.32±0.08	0.05±0.00
**A-15**	**5.25±0.57**	**3.32±0.42**	**24.09±1.80**	**21.56±2.99**	**4.32±0.15**
	0.15±0.02	0.10±0.01	0.69±0.06	0.62±0.09	0.13±0.00
**A-16**	**3.39±0.71**	**4.25±0.40**	**23.26±0.02**	**17.68±0.87**	**2.75±0.32**
	0.12±0.03	0.15±0.01	0.83±0.02	0.63±0.02	0.10±0.01
**A-17**	**2.50±0.49**	**3.02±0.50**	**19.96±1.68**	**13.79±2.60**	**2.42±0.30**
	0.09±0.02	0.11±0.02	0.72±0.10	0.50±0.12	0.09±0.02
**A-18**	**3.23±0.48**	**2.92±0.02**	**30.31±0.29**	**29.43±2.75**	**3.29±0.66**
	0.09±0.01	0.08±0.01	0.87±0.04	0.83±0.04	0.09±0.01
**A-19**	**2.51±0.09**	**2.69±0.06**	**20.65±3.48**	**13.65±0.38**	**1.95±0.02**
	0.07±0.01	0.08±0.01	0.65±0.08	0.39±0.02	0.06±0.00
**A-20**	**4.46±0.18**	**4.18±0.82**	**37.77±0.62**	**32.87±5.73**	**4.02±0.86**
	0.05±0.00	0.04±0.01	0.40±0.01	0.35±0.06	0.04±0.01
**A-21**	**3.92±0.71**	**3.55±0.12**	**38.30±2.28**	**36.50±1.56**	**3.54±0.30**
	0.04±0.01	0.04±0.00	0.43±0.02	0.41±0.02	0.04±0.00
**A-22**	**2.29±0.38**	**3.08±0.34**	**21.93±3.18**	**26.26±1.15**	**2.60±0.48**
	0.07±0.01	0.09±0.01	0.67±0.08	0.78±0.02	0.08±0.01
**A-23**	**2.58±0.46**	**2.00±0.19**	**18.27±0.08**	**13.68±2.72**	**2.59±0.42**
	0.08±0.01	0.06±0.01	0.58±0.01	0.44±0.09	0.08±0.01
**B-24**	**4.26±0.39**	**5.61±0.08**	**28.58±1.47**	**20.60±0.79**	**3.68±0.30**
	0.13±0.01	0.17±0.01	0.85±0.06	0.62±0.01	0.11±0.01
**B-25**	**3.67±0.06**	**4.46±0.48**	**29.04±0.69**	**21.56±0.82**	**4.76±0.31**
	0.09±0.00	0.12±0.00	0.75±0.01	0.56±0.00	0.12±0.00
**B-26**	**3.54±0.66**	**3.73±0.34**	**29.46±0.59**	**21.99±1.73**	**2.61±0.49**
	0.07±0.02	0.08±0.00	0.61±0.04	0.46±0.07	0.06±0.01
**B-27**	**2.90±0.20**	**3.74±0.13**	**22.06±1.39**	**16.28±0.38**	**2.84±0.23**
	0.06±0.01	0.07±0.01	0.44±0.06	0.33±0.02	0.06±0.00
**B-28**	**3.88±0.43**	**4.59±0.74**	**25.92±4.48**	**17.52±2.94**	**2.68±0.18**
	0.08±0.00	0.10±0.01	0.55±0.04	0.37±0.03	0.06±0.01
**B-29**	**4.00±0.37**	**5.28±0.20**	**33.63±1.29**	**22.32±3.22**	**3.60±0.60**
	0.08±0.00	0.10±0.01	0.66±0.00	0.44±0.05	0.07±0.01
**B-30**	**4.82±1.03**	**5.58±0.14**	**28.57±2.27**	**17.41±2.95**	**3.66±0.75**
	0.09±0.01	0.10±0.02	0.53±0.03	0.33±0.03	0.06±0.00
**B-31**	**4.93±0.44**	**7.00±0.38**	**43.54±3.96**	**43.49±10.05**	**12.36±0.93**
	0.02±0.00	0.03±0.00	0.21±0.00	0.21±0.03	0.06±0.01
**B-32**	**4.44±0.30**	**4.76±0.05**	**77.50±12.32**	**75.46±11.01**	**5.20±0.41**
	0.03±0.01	0.03±0.00	0.42±0.05	0.41±0.05	0.03±0.00
**B-33**	**6.31±0.67**	**4.62±0.87**	**40.38±7.66**	**23.79±4.69**	**4.98±0.88**
	0.05±0.00	0.03±0.00	0.32±0.02	0.19±0.02	0.04±0.00

**B-34**	**3.68±0.48**	**4.75±0.24**	**36.28±1.25**	**25.73±1.49**	**6.10±0.11**
	0.03±0.00	0.04±0.00	0.34±0.02	0.24±0.02	0.06±0.00
**B-35**	**3.40±0.21**	**3.49±0.42**	**26.95±2.55**	**19.56±0.41**	**2.91±0.21**
	0.11±0.00	0.11±0.01	0.85±0.04	0.62±0.01	0.09±0.01
**B-36**	**3.62±0.05**	**5.21±0.18**	**22.83±3.07**	**16.86±1.52**	**3.35±0.25**
	0.12±0.00	0.17±0.02	0.74±0.05	0.55±0.02	0.11±0.01
**B-37**	**3.22±0.24**	**4.07±0.82**	**29.17±5.16**	**21.65±0.31**	**3.05±0.20**
	0.11±0.00	0.13±0.02	0.88±0.16	0.65±0.01	0.10±0.00
**B-38**	**4.39±0.15**	**4.87±0.72**	**36.23±0.01**	**25.89±0.83**	**3.10±0.10**
	0.09±0.00	0.10±0.01	0.76±0.02	0.54±0.00	0.07±0.00
**C-39**	**5.00±0.22**	**5.58±0.76**	**34.51±2.12**	**30.95±4.99**	**5.04±0.32**
	0.10±0.00	0.11±0.01	0.71±0.05	0.64±0.09	0.10±0.01
**C-40**	**3.46±0.65**	**4.24±0.83**	**29.63±4.59**	**22.83±5.01**	**3.35±0.29**
	0.06±0.01	0.07±0.00	0.63±0.11	0.51±0.11	0.07±0.01
**C-41**	**4.92±0.39**	**5.91±0.72**	**36.65±1.69**	**27.76±0.75**	**3.99±0.10**
	0.11±0.02	0.13±0.01	0.79±0.02	0.60±0.03	0.09±0.01
**C-42**	**5.67±0.48**	**7.77±0.41**	**32.80±0.64**	**26.38±2.09**	**5.63±0.62**
	0.12±0.02	0.16±0.02	0.68±0.05	0.55±0.08	0.12±0.02
**C-43**	**5.31±1.11**	**6.88±0.49**	**33.86±3.28**	**27.48±3.05**	**6.27±1.21**
	0.11±0.03	0.15±0.01	0.72±0.09	0.58±0.08	0.13±0.02
**C-44**	**6.92±0.86**	**6.63±0.01**	**54.19±2.29**	**38.47±3.35**	**6.58±0.39**
	0.07±0.00	0.07±0.00	0.54±0.02	0.38±0.01	0.07±0.00
**C-45**	**5.14±0.99**	**6.40±0.62**	**39.98±7.45**	**27.54±3.47**	**4.42±0.07**
	0.10±0.02	0.13±0.01	0.78±0.12	0.54±0.05	0.09±0.00
**C-46**	**5.22±0.50**	**5.89±0.89**	**37.50±1.96**	**26.04±3.56**	**6.15±0.62**
	0.11±0.00	0.13±0.03	0.79±0.05	0.54±0.02	0.13±0.00
**C-47**	**4.35±0.14**	**3.88±0.12**	**32.09±0.38**	**26.73±1.70**	**4.25±0.32**
	0.09±0.01	0.08±0.00	0.65±0.03	0.54±0.00	0.09±0.00
**C-48**	**5.03±0.80**	**7.09±1.28**	**36.92±6.49**	**26.09±1.10**	**9.52±0.85**
	0.11±0.01	0.15±0.02	0.77±0.10	0.55±0.00	0.20±0.01
**D-49**	**3.91±0.93**	**3.12±0.70**	**29.98±4.42**	**25.38±5.17**	**3.66±0.75**
	0.16±0.01	0.13±0.01	1.28±0.08	1.04±0.09	0.15±0.02
**D-50**	**4.38±0.83**	**4.02±0.42**	**27.66±3.46**	**20.91±3.49**	**3.11±0.60**
	0.20±0.00	0.18±0.02	1.27±0.09	0.95±0.03	0.14±0.00
**D-51**	**4.15±0.56**	**3.77±0.20**	**30.10±1.93**	**20.30±0.23**	**2.25±0.24**
	0.18±0.00	0.14±0.01	1.12±0.05	0.76±0.09	0.08±0.02
**D-52**	**3.66±0.37**	**4.30±0.18**	**22.28±1.92**	**17.04±0.79**	**2.63±0.11**
	0.13±0.01	0.15±0.00	0.78±0.05	0.60±0.01	0.09±0.00
**D-53**	**4.64±0.40**	**4.85±0.39**	**38.47±2.56**	**31.88±3.71**	**3.46±0.16**
	0.03±0.00	0.04±0.00	0.29±0.01	0.24±0.03	0.03±0.00
**D-54**	**4.29±0.19**	**4.32±0.34**	**29.12±2.28**	**19.66±0.34**	**3.12±0.27**
	0.15±0.01	0.15±0.01	1.00±0.08	0.68±0.01	0.11±0.01
**D-55**	**4.15±0.47**	**4.92±0.22**	**24.18±2.81**	**15.94±0.86**	**2.84±0.00**
	0.14±0.01	0.17±0.00	0.82±0.08	0.54±0.02	0.10±0.00
**D-56**	**3.94±0.67**	**4.71±0.55**	**26.04±1.70**	**19.70±2.09**	**2.85±0.19**
	0.15±0.01	0.17±0.00	0.97±0.04	0.73±0.00	0.11±0.00
**D-57**	**4.12±0.32**	**3.50±0.55**	**37.34±2.34**	**27.17±0.69**	**3.26±0.34**
	0.13±0.02	0.11±0.01	1.15±0.00	0.84±0.08	0.10±0.00
**D-58**	**3.41±0.12**	**2.49±0.07**	**28.67±0.41**	**21.16±0.61**	**2.90±0.03**
	0.11±0.01	0.08±0.01	0.90±0.03	0.66±0.05	0.09±0.00
**E-59**	**4.25±0.01**	**5.74±0.64**	**26.72±1.82**	**20.27±0.03**	**6.28±1.24**
	0.13±0.01	0.17±0.04	0.80±0.13	0.61±0.06	0.19±0.02
**E-60**	**4.43±0.35**	**5.42±0.78**	**28.30±2.94**	**20.33±2.66**	**4.55±0.18**
	0.12±0.01	0.15±0.02	0.79±0.08	0.57±0.08	0.13±0.00
**E-61**	**4.46±0.25**	**5.73±0.87**	**28.60±4.24**	**22.64±0.44**	**2.79±0.12**
	0.13±0.01	0.16±0.03	0.82±0.14	0.65±0.03	0.08±0.00
**E-62**	**5.16±0.09**	**6.79±0.18**	**30.70±1.87**	**22.63±4.35**	**5.89±0.72**
	0.15±0.02	0.20±0.04	0.76±0.04	0.63±0.05	0.17±0.01
**E-63**	**4.34±0.48**	**6.70±1.17**	**29.20±2.00**	**21.49±2.05**	**4.54±0.15**
	0.12±0.00	0.19±0.01	0.82±0.03	0.60±0.01	0.13±0.01
**E-64**	**4.91±0.04**	**5.20±0.15**	**32.38±4.21**	**23.72±3.52**	**4.60±0.01**
	0.13±0.01	0.14±0.00	0.86±0.06	0.64±0.07	0.12±0.01
**E-65**	**5.05±0.21**	**5.98±0.52**	**35.05±3.56**	**23.92±0.31**	**3.74±0.27**
	0.14±0.00	0.17±0.01	1.00±0.06	0.55±0.06	0.10±0.01

Values are expressed as mean ± SD (n=4). Legend: CSA*n*: caffeoylshikimic acid isomer; CQL*n*: caffeoylquinic lactone isomer; *n*-FQL: *n*-*O*-feruloylquinic lactone.

SUPPLEMENTARY FIGURE CAPTION

Supplementary Figure 1. Loading plot (A) and score plot (B) obtained from the PCA with varimax of the considered bioactive compounds and capsule espresso coffees, by using bioactive concentrations (mg/mL). Legend: *n*-CQA: *n*-*O*-caffeoylquinic acid; CSA*n*: caffeoylshikimic acid isomer; CQL*n*: caffeoylquinic lactone isomer; *n*-CouQA: *n*-*O*-coumaroylquinic acid; *n*-FQA: *n*-*O*-feruloylquinic acid; *n*-FQL: *n*-*O*-feruloylquinic lactone; NMP: *N*-methylpyridinium.

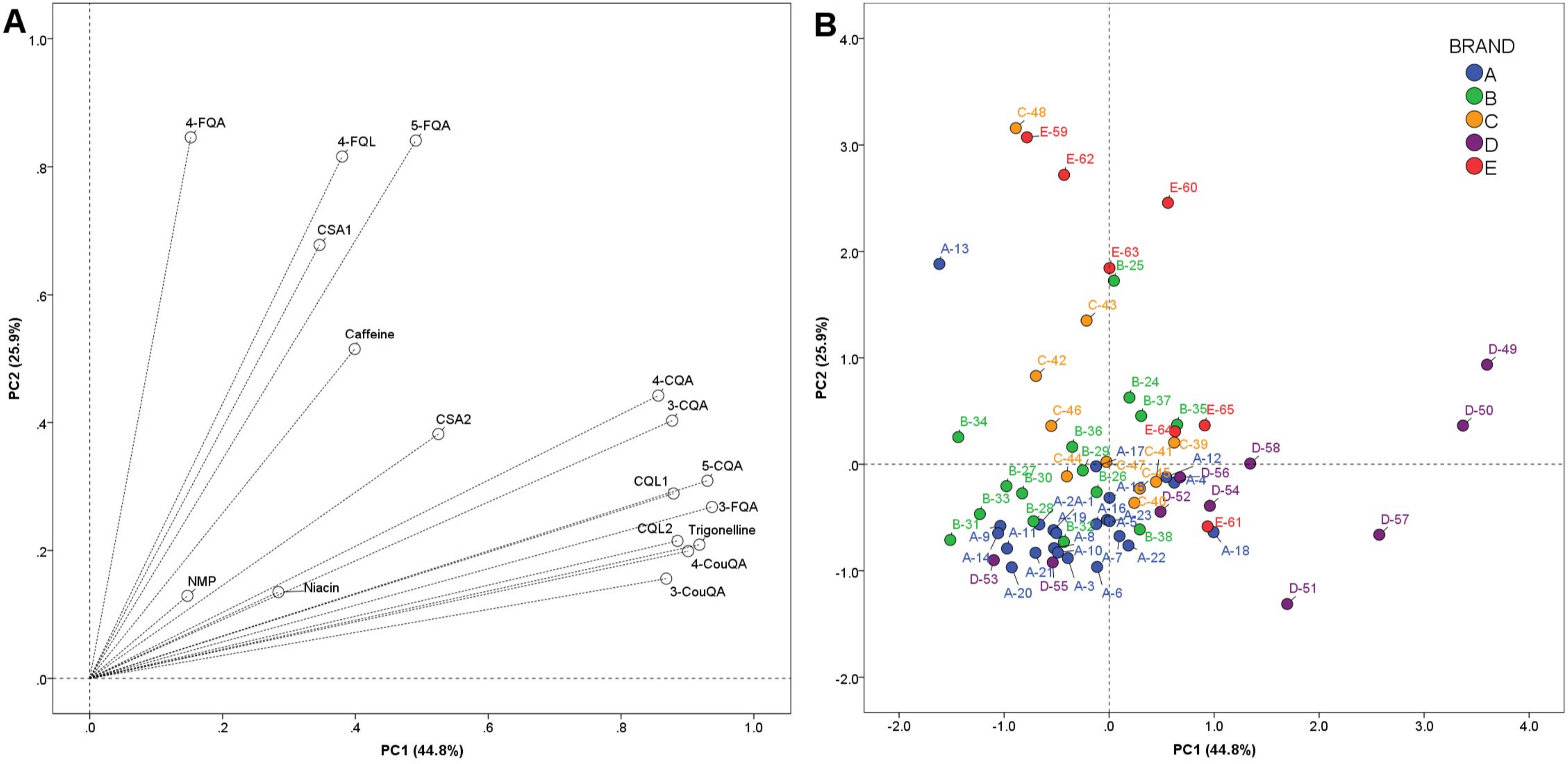
A
4-FQA
4-FQL
5-FQA
CSA1
Caffeine
CSA2
4-CQA
3-CQA
5-CQA
CQL1
3-FQA
CQL2
Trigonelline
4-CouQA
NMP
Niacin
3-CouQA
PC2 (25.9%)
PC1 (44.8%)
B
BRAND
A
B
C
D
E
C-48
E-59
E-62
E-60
A-13
E-63
B-25
C-43
C-42
D-49
B-24
B-37
B-35
E-65
D-50
C-46
B-34
E-64
B-36
C-39
D-58
B-29
A-17
C-41
C-44
A-12
B-27
B-30
B-26
D-56
A-4
D-54
B-33
D-52
B-28
A-16
A-23
B-31
A-11
A-9
A-19
A-5
B-38
E-61
D-57
A-8
A-10
A-18
A-14
A-7
A-22
A-21
D-53
D-55
A-3
A-6
A-20
D-51
PC2 (25.9%)
PC1 (44.8%)
